# Supplementary figures and images for: Bayesian analysis of Formula One race results: disentangling driver skill and constructor advantage
Source: J Quant Anal Sports. 2023 Jul 25;19(4):273–93. doi: 10.1515/jqas-2022-0021 (PMC10660124; doi:10.1515/jqas-2022-0021)

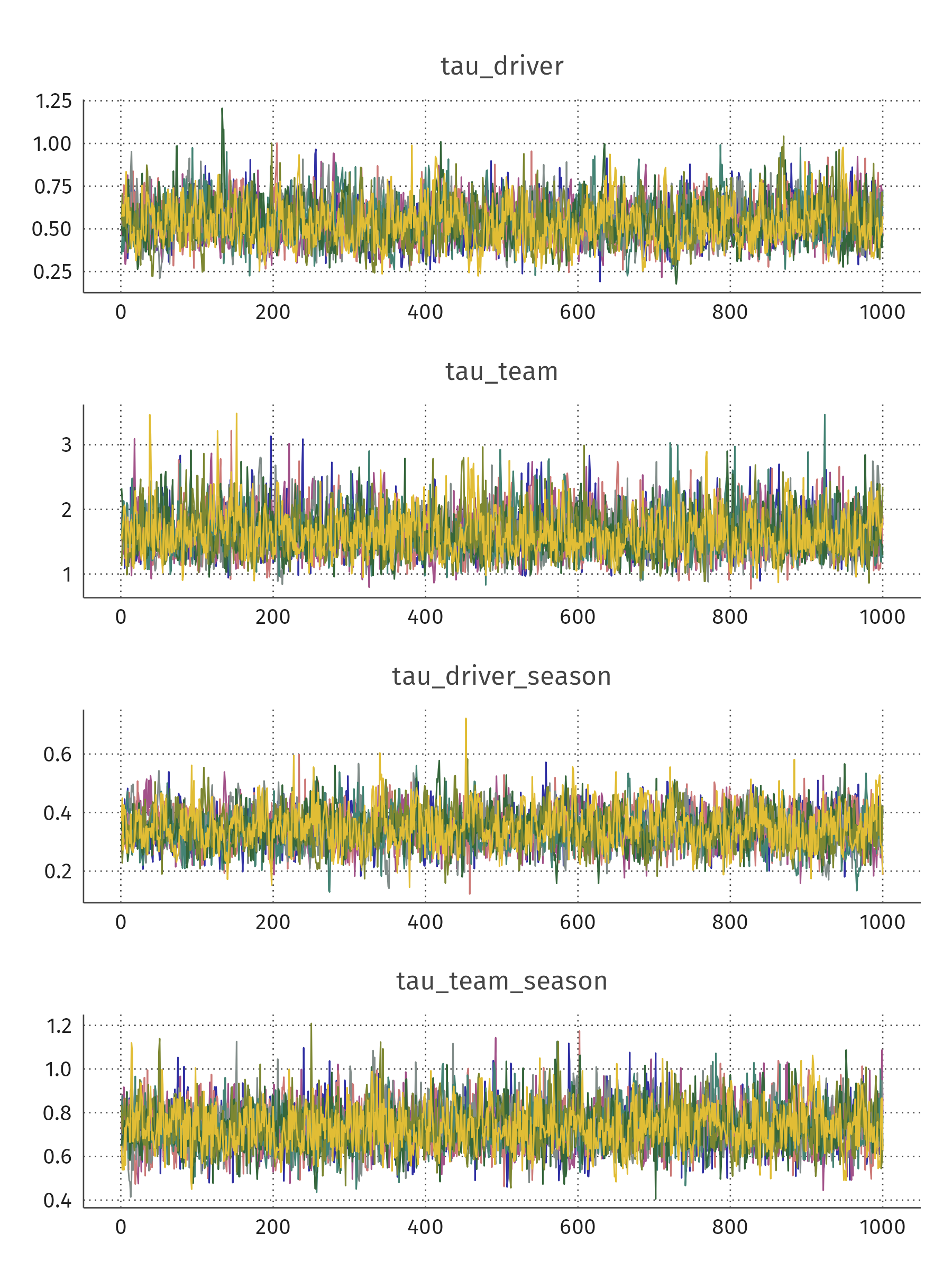

Supplement: Supplementary file 1 — Supplementary Material Details [file j_jqas-2022-0021_suppl_001.zip › img/chains.png]

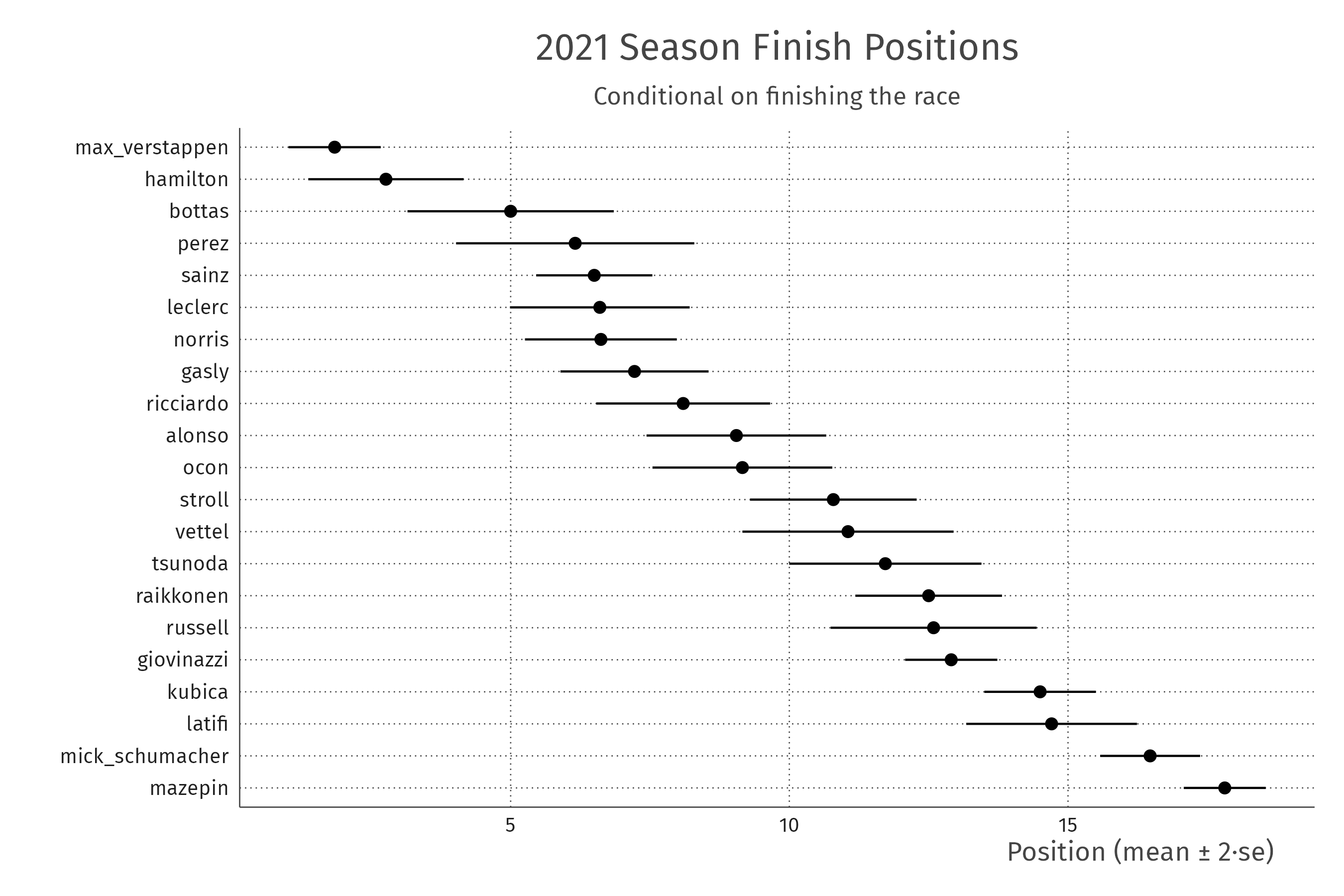

Supplement: Supplementary file 1 — Supplementary Material Details [file j_jqas-2022-0021_suppl_001.zip › img/eda_finish_2021.png]

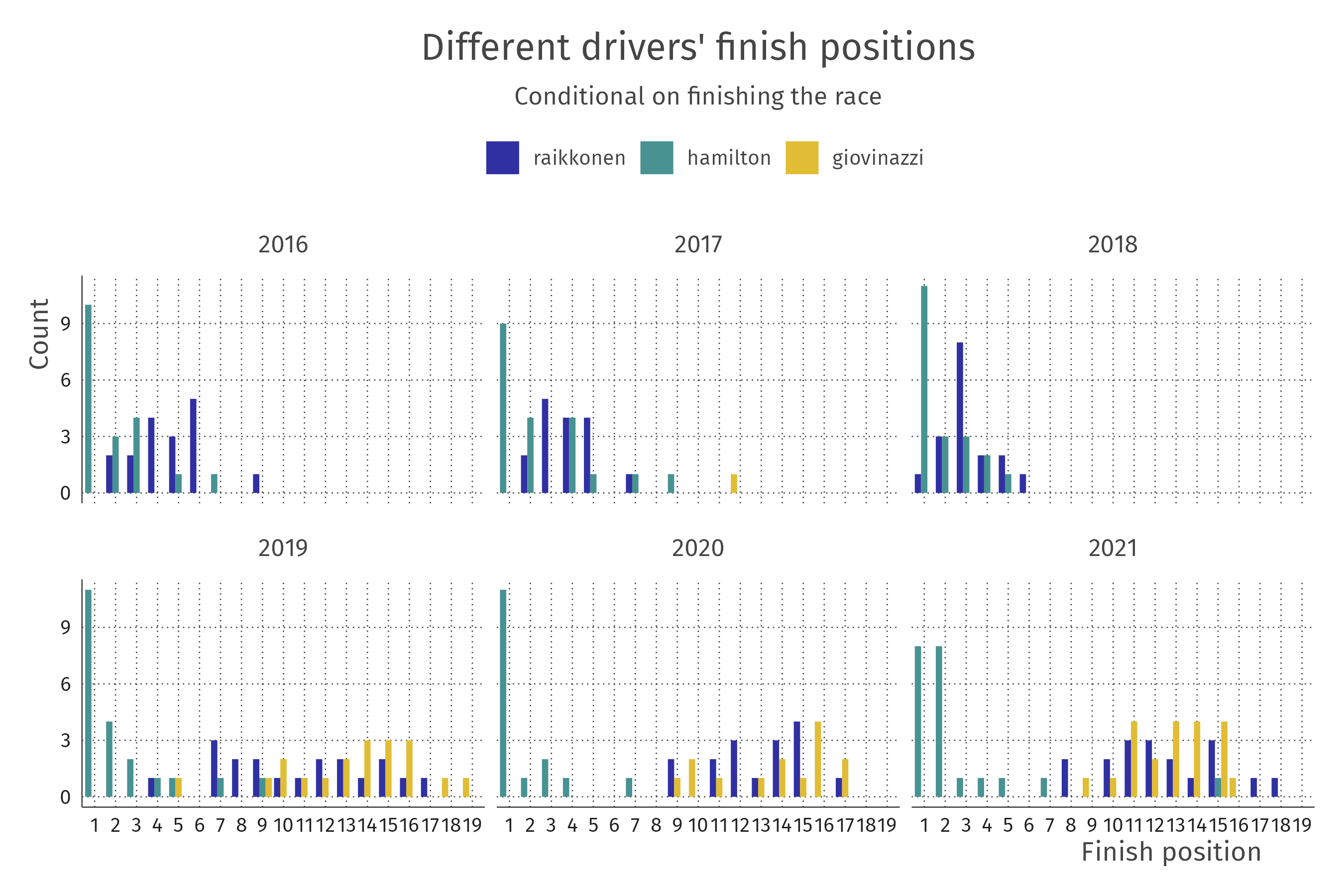

Supplement: Supplementary file 1 — Supplementary Material Details [file j_jqas-2022-0021_suppl_001.zip › img/eda_finish_drivers.png]

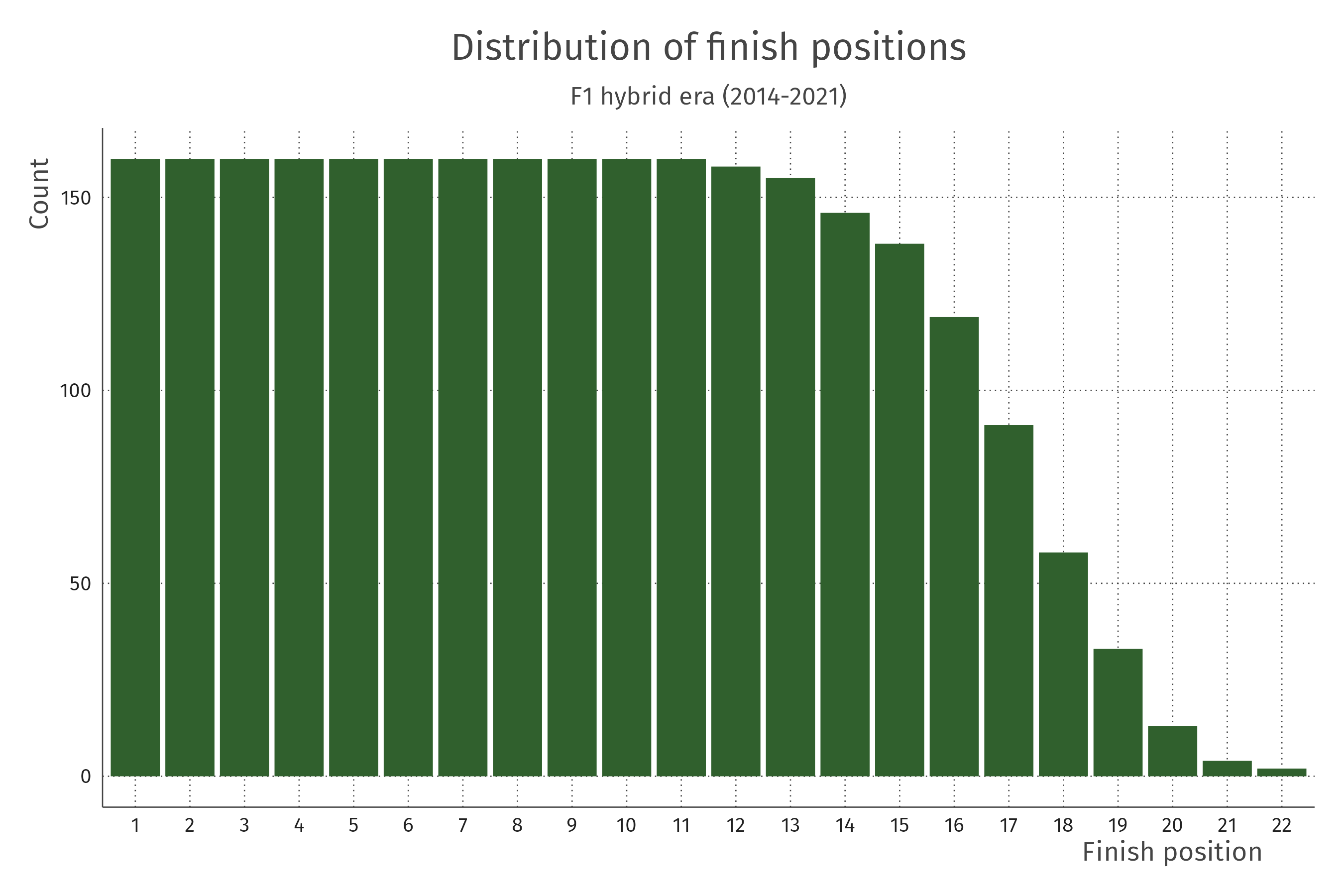

Supplement: Supplementary file 1 — Supplementary Material Details [file j_jqas-2022-0021_suppl_001.zip › img/eda_finish_position.png]

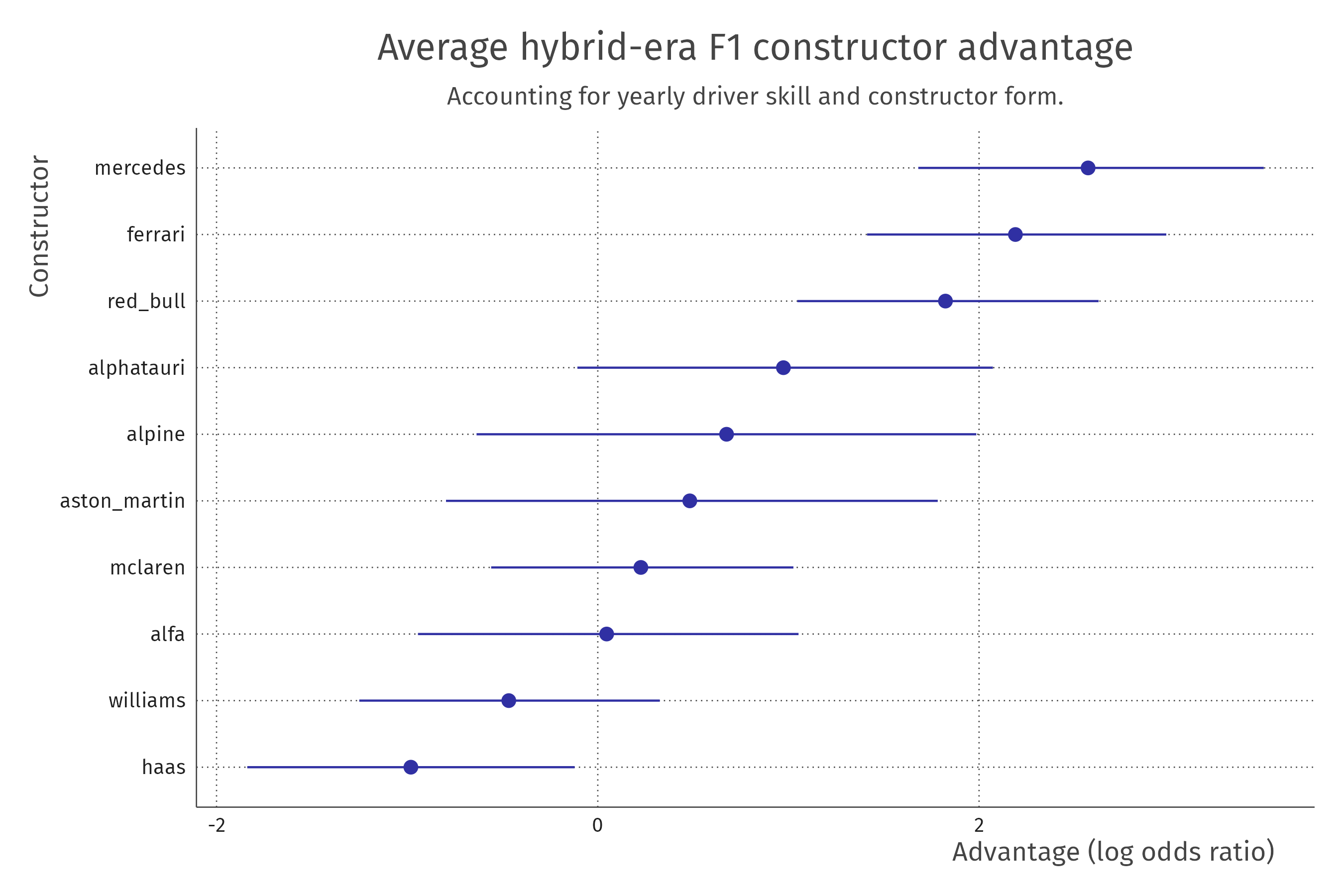

Supplement: Supplementary file 1 — Supplementary Material Details [file j_jqas-2022-0021_suppl_001.zip › img/plt_advantage_avg.png]

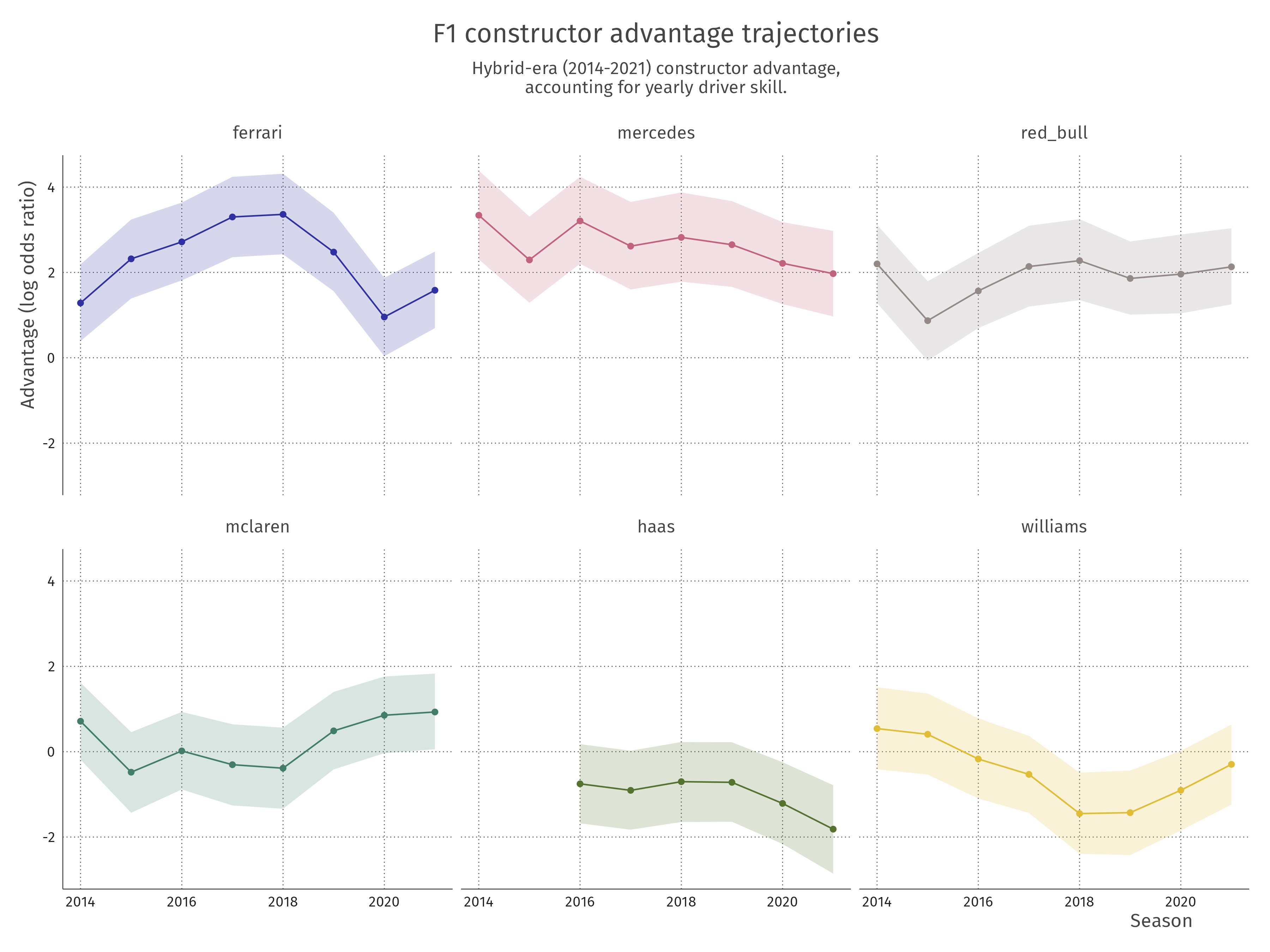

Supplement: Supplementary file 1 — Supplementary Material Details [file j_jqas-2022-0021_suppl_001.zip › img/plt_advantage_trajectory.png]

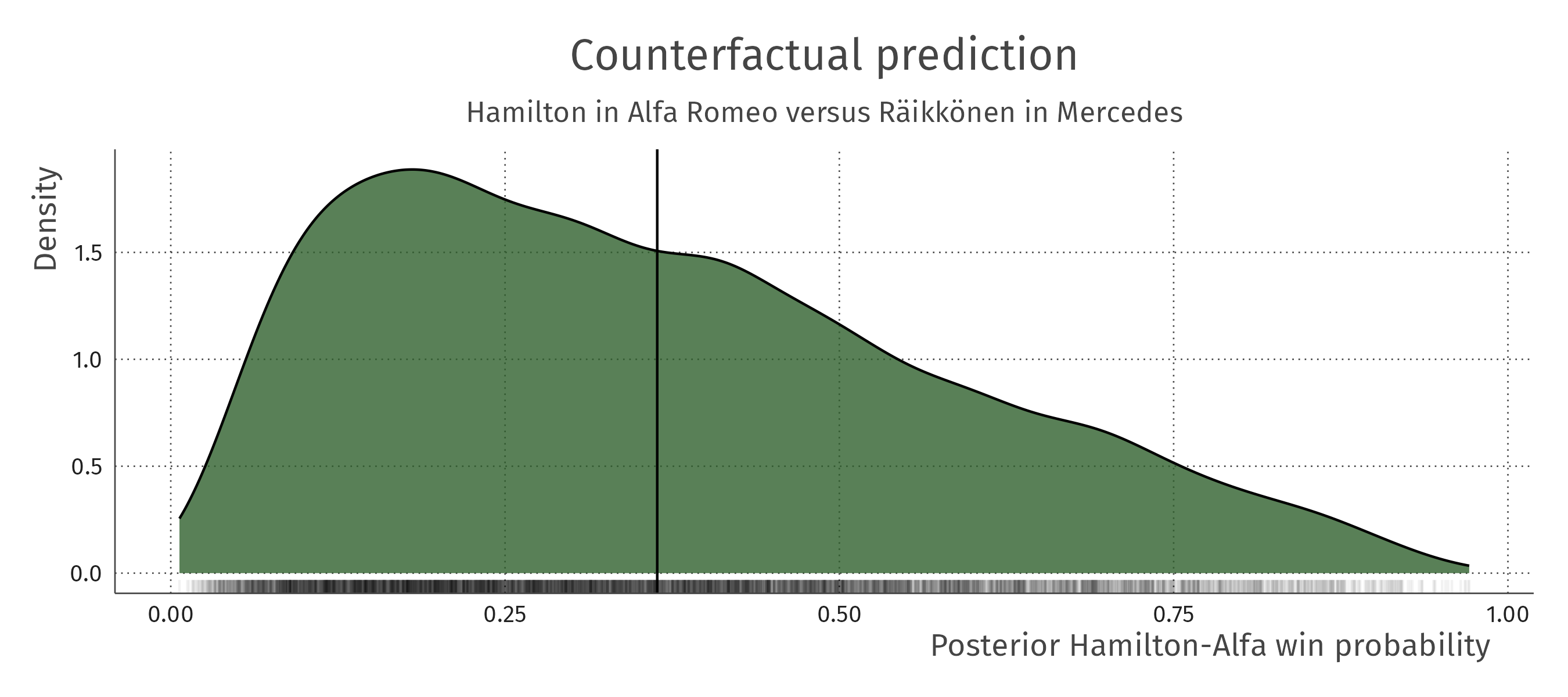

Supplement: Supplementary file 1 — Supplementary Material Details [file j_jqas-2022-0021_suppl_001.zip › img/plt_counterfactual.png]

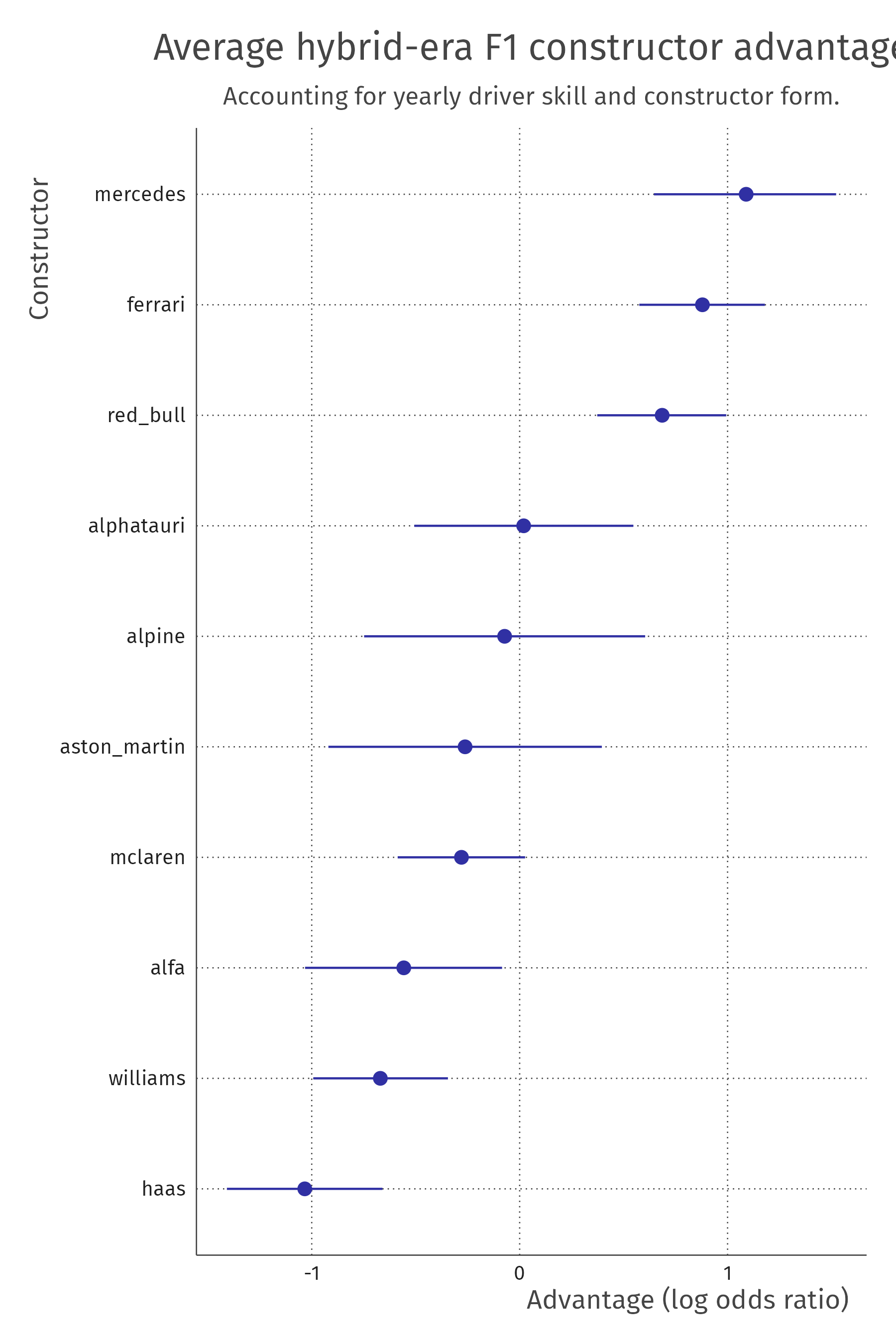

Supplement: Supplementary file 1 — Supplementary Material Details [file j_jqas-2022-0021_suppl_001.zip › img/plt_performance_2021.png]

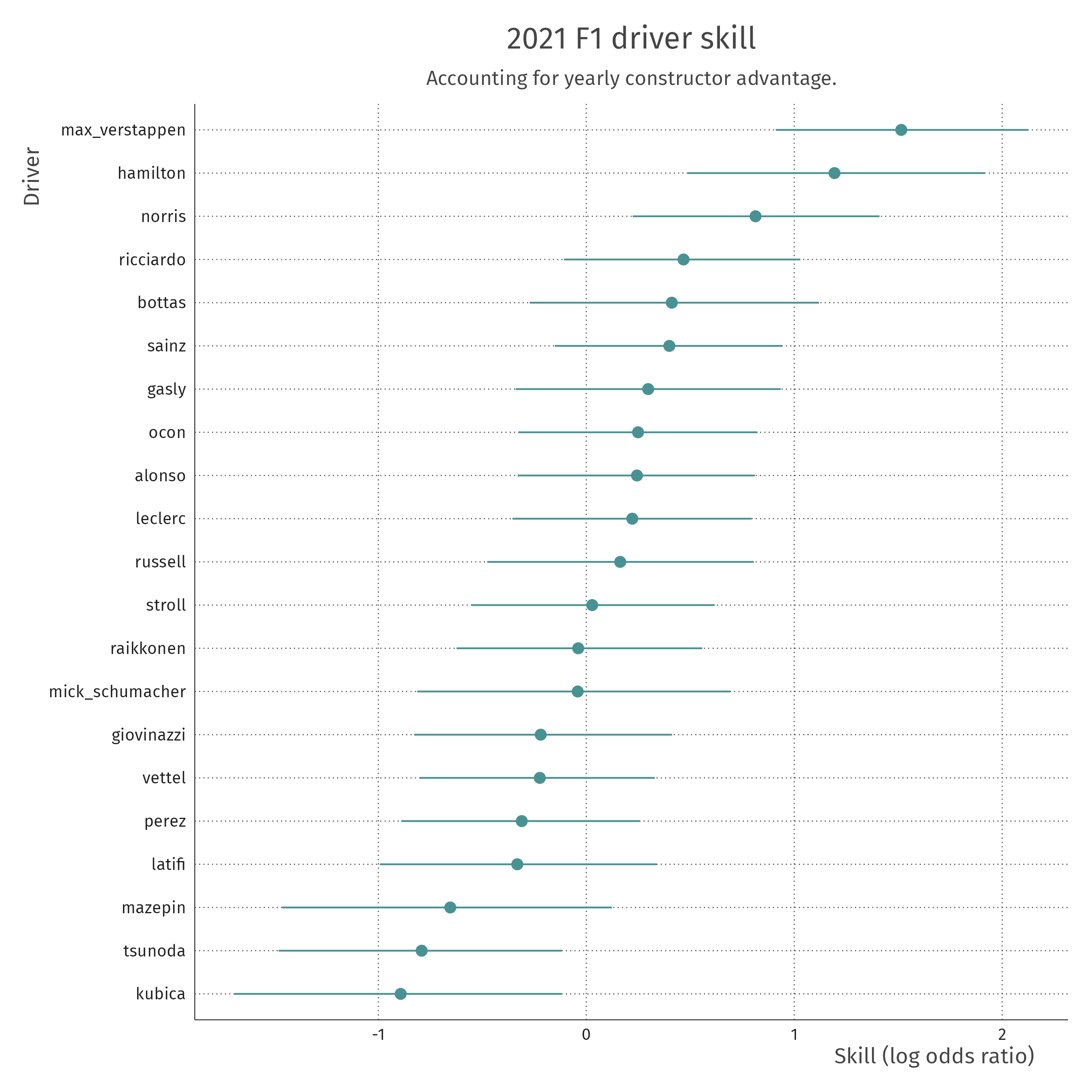

Supplement: Supplementary file 1 — Supplementary Material Details [file j_jqas-2022-0021_suppl_001.zip › img/plt_skill_2021.png]

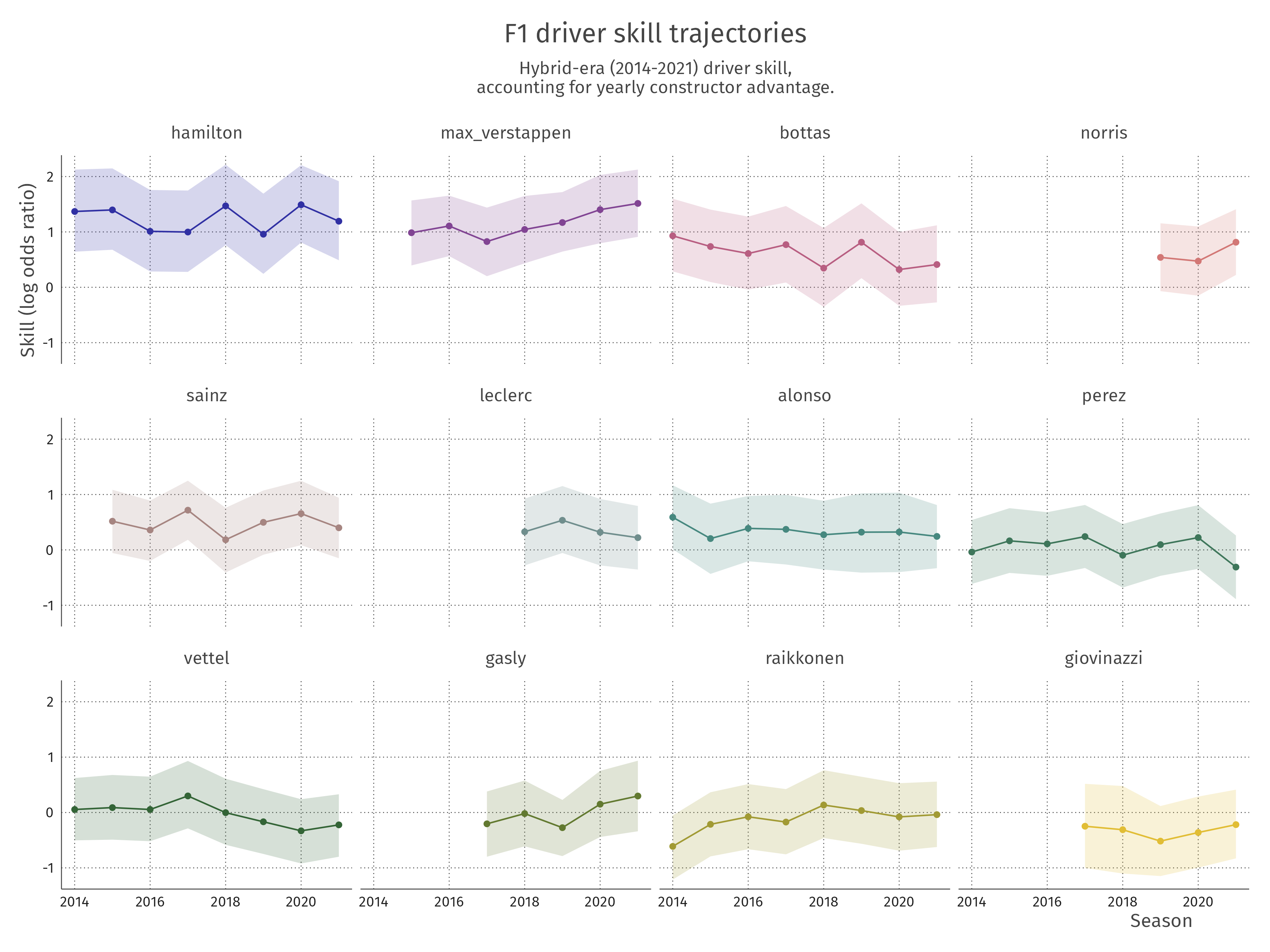

Supplement: Supplementary file 1 — Supplementary Material Details [file j_jqas-2022-0021_suppl_001.zip › img/plt_skill_trajectories.png]

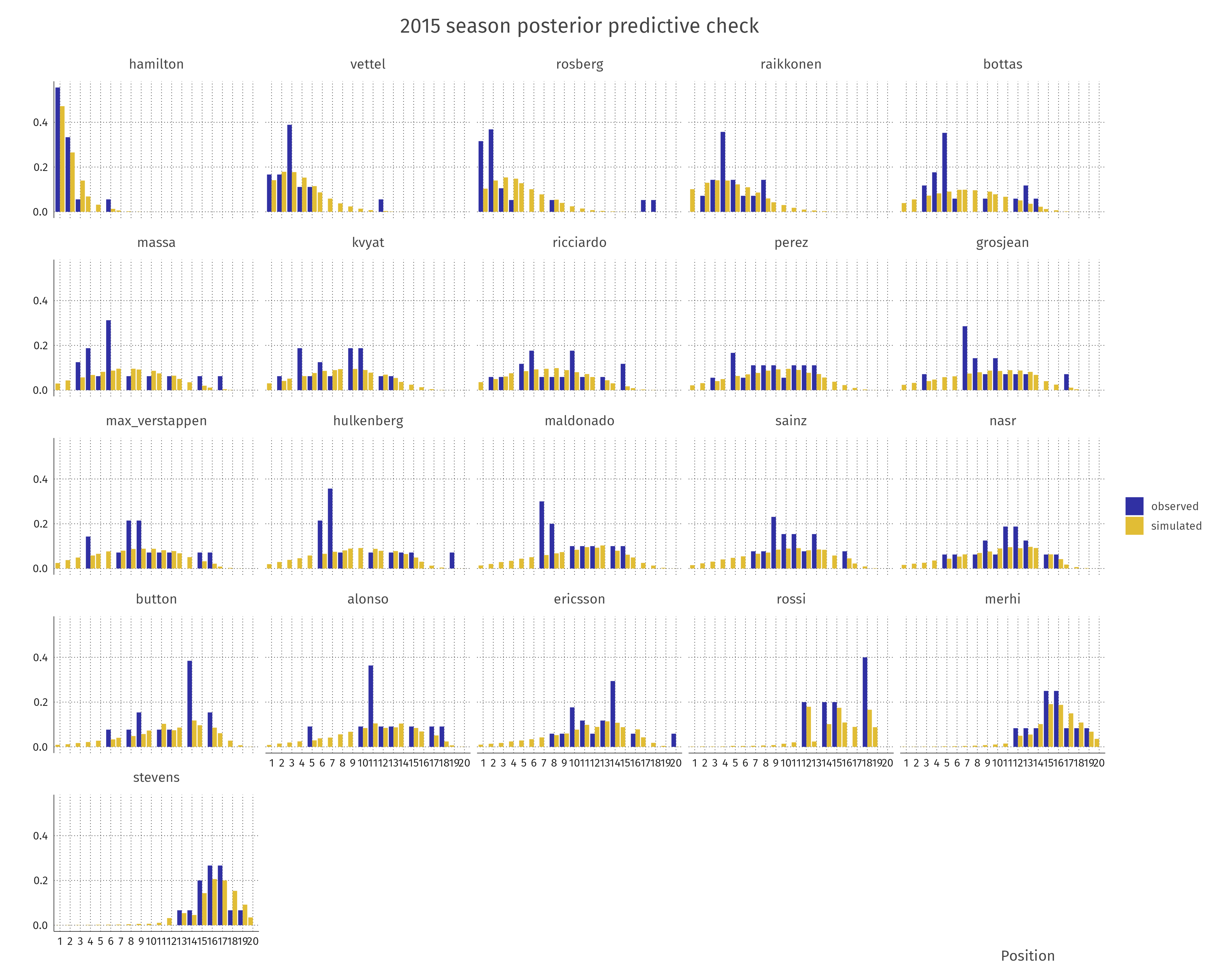

Supplement: Supplementary file 1 — Supplementary Material Details [file j_jqas-2022-0021_suppl_001.zip › img/pp_check_rank_2015.png]

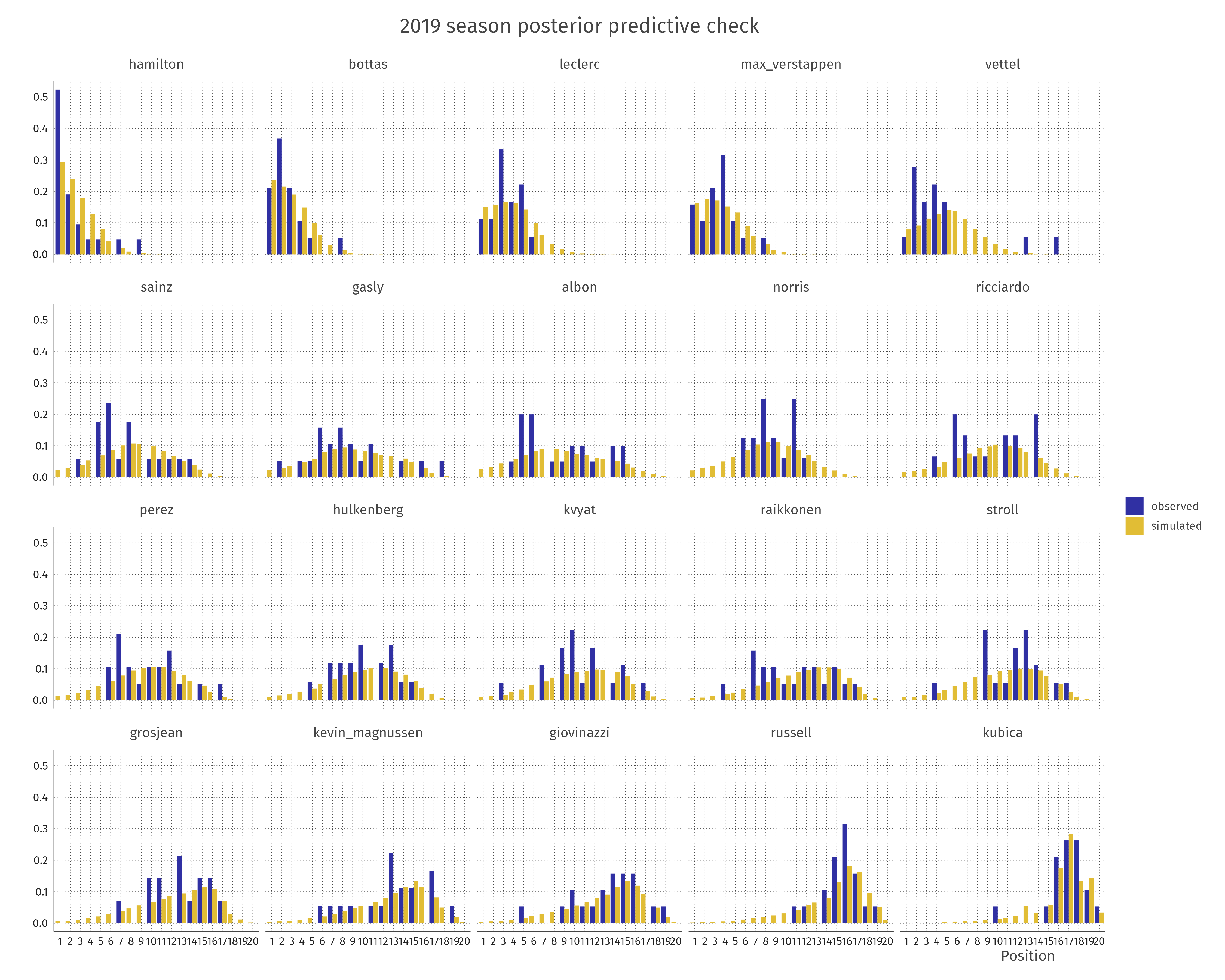

Supplement: Supplementary file 1 — Supplementary Material Details [file j_jqas-2022-0021_suppl_001.zip › img/pp_check_rank_2019.png]

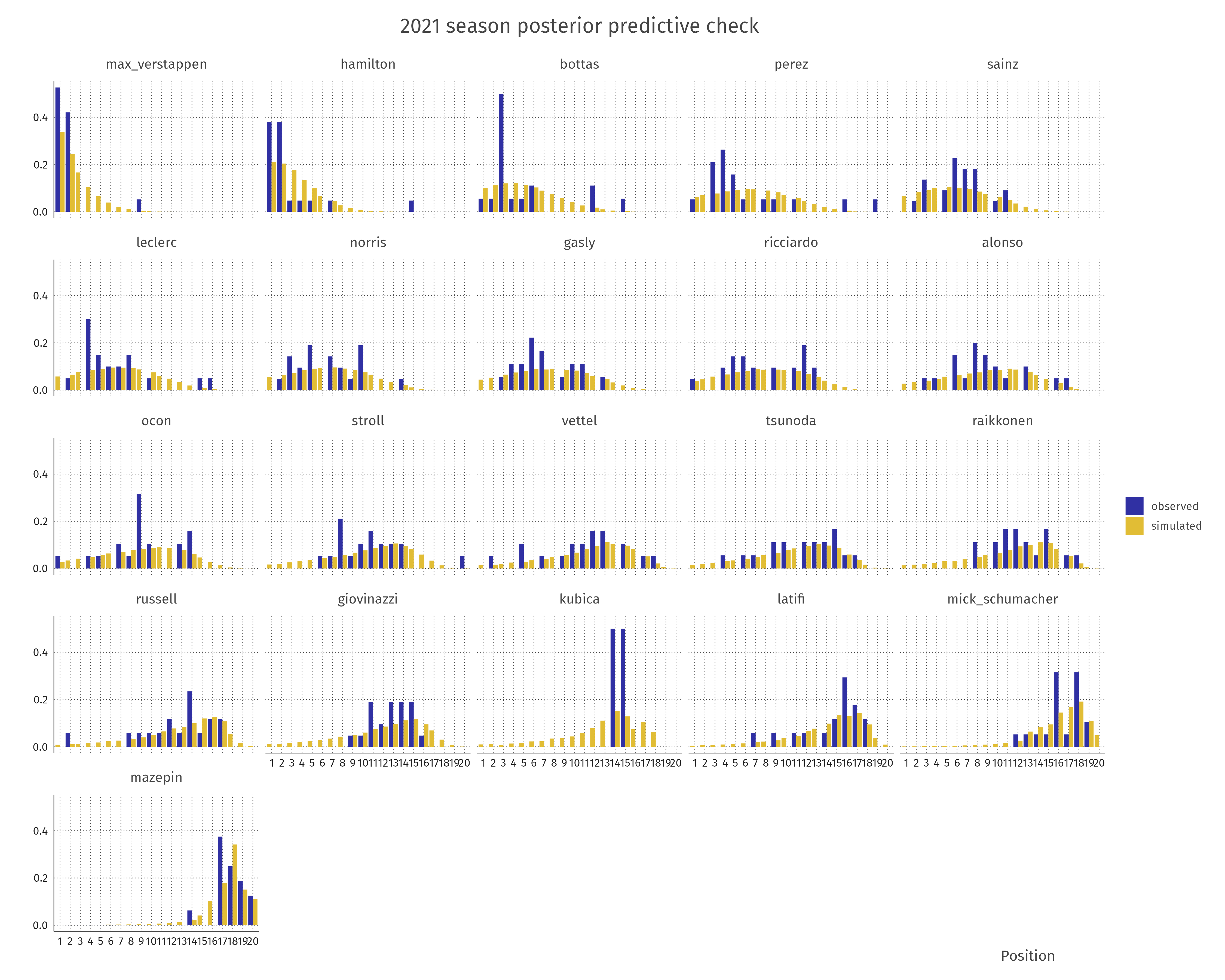

Supplement: Supplementary file 1 — Supplementary Material Details [file j_jqas-2022-0021_suppl_001.zip › img/pp_check_rank_2021.png]

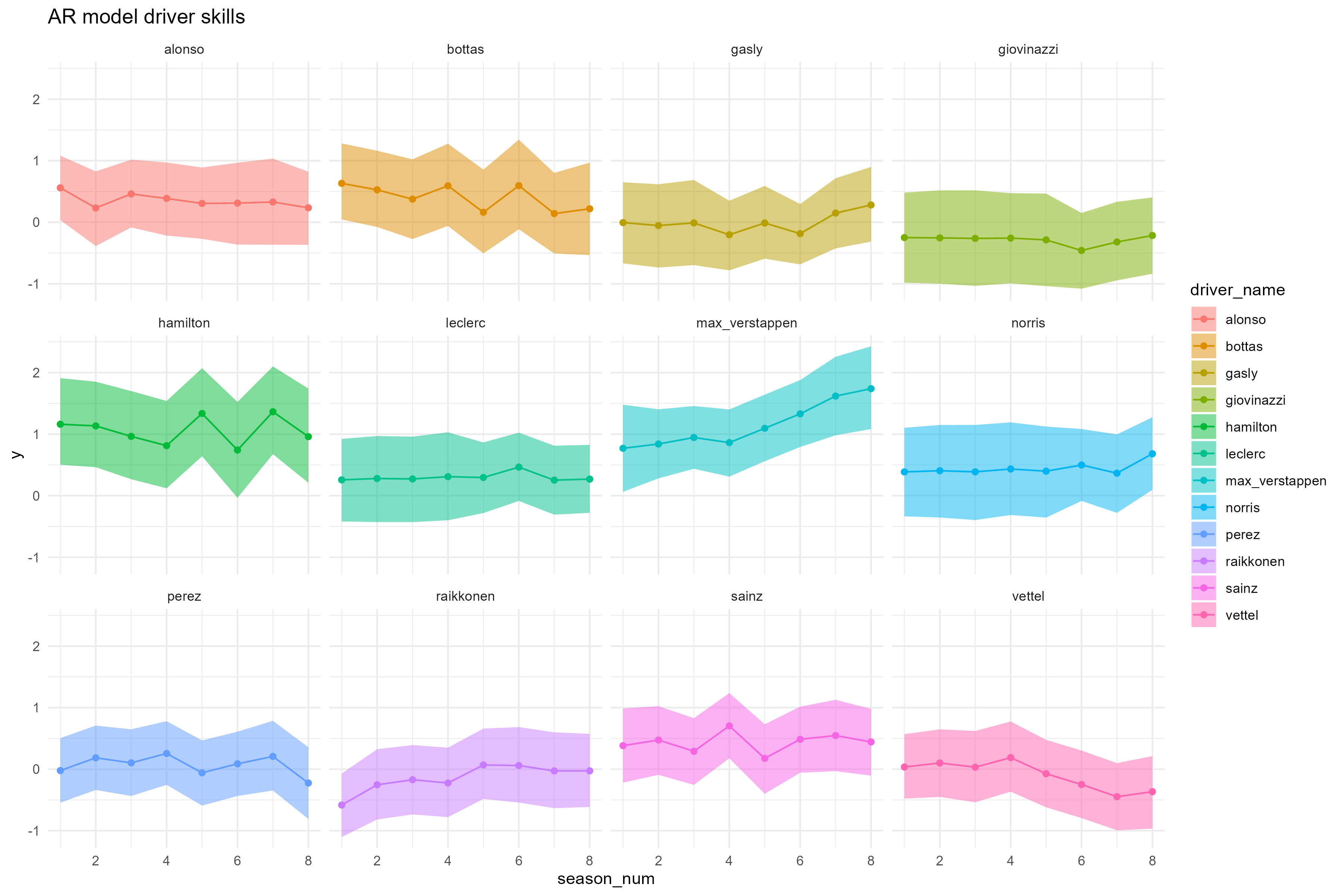

Supplement: Supplementary file 1 — Supplementary Material Details [file j_jqas-2022-0021_suppl_001.zip › model_comparison/img/ar_driver.png]

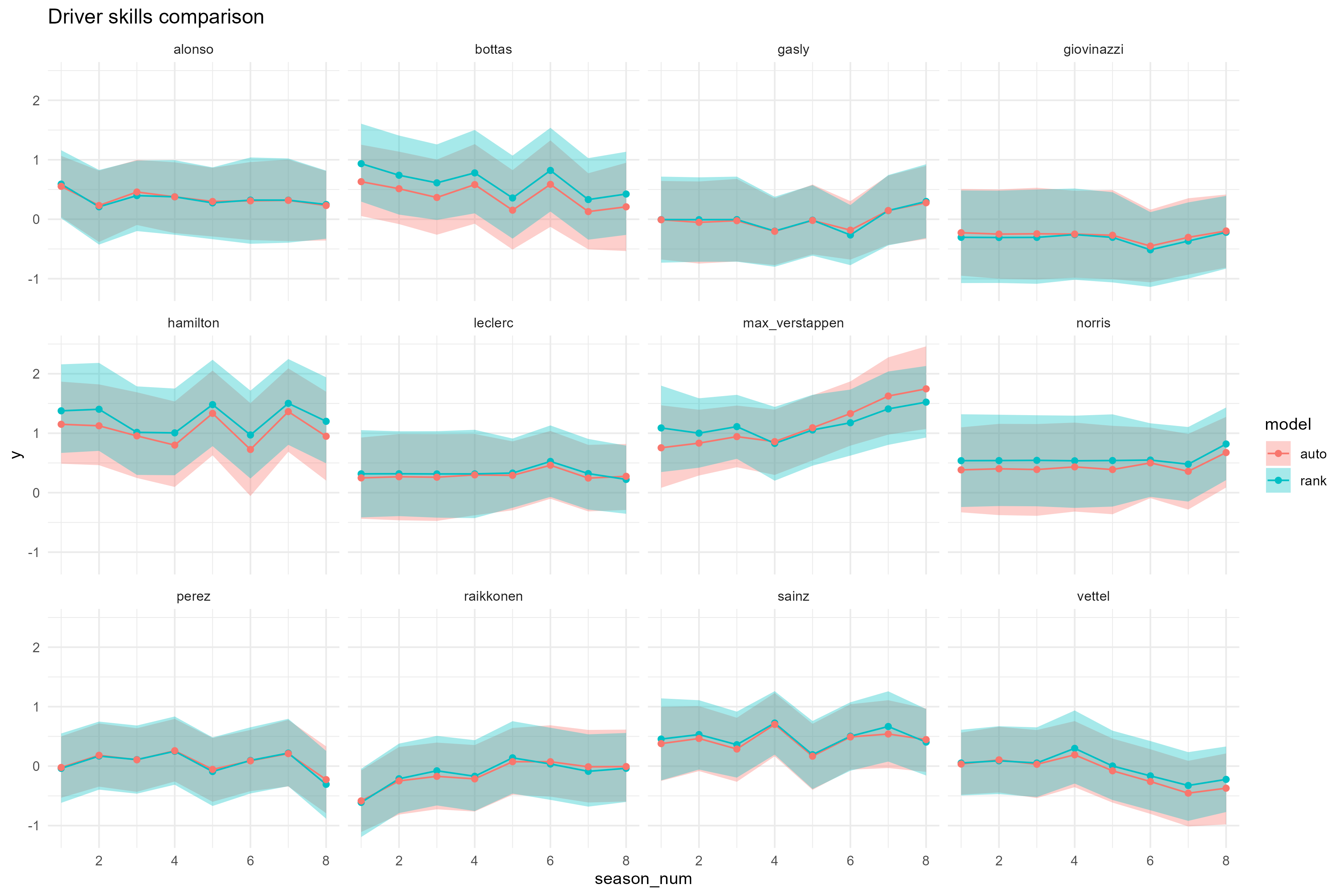

Supplement: Supplementary file 1 — Supplementary Material Details [file j_jqas-2022-0021_suppl_001.zip › model_comparison/img/ar_rank_driver_comparison.png]

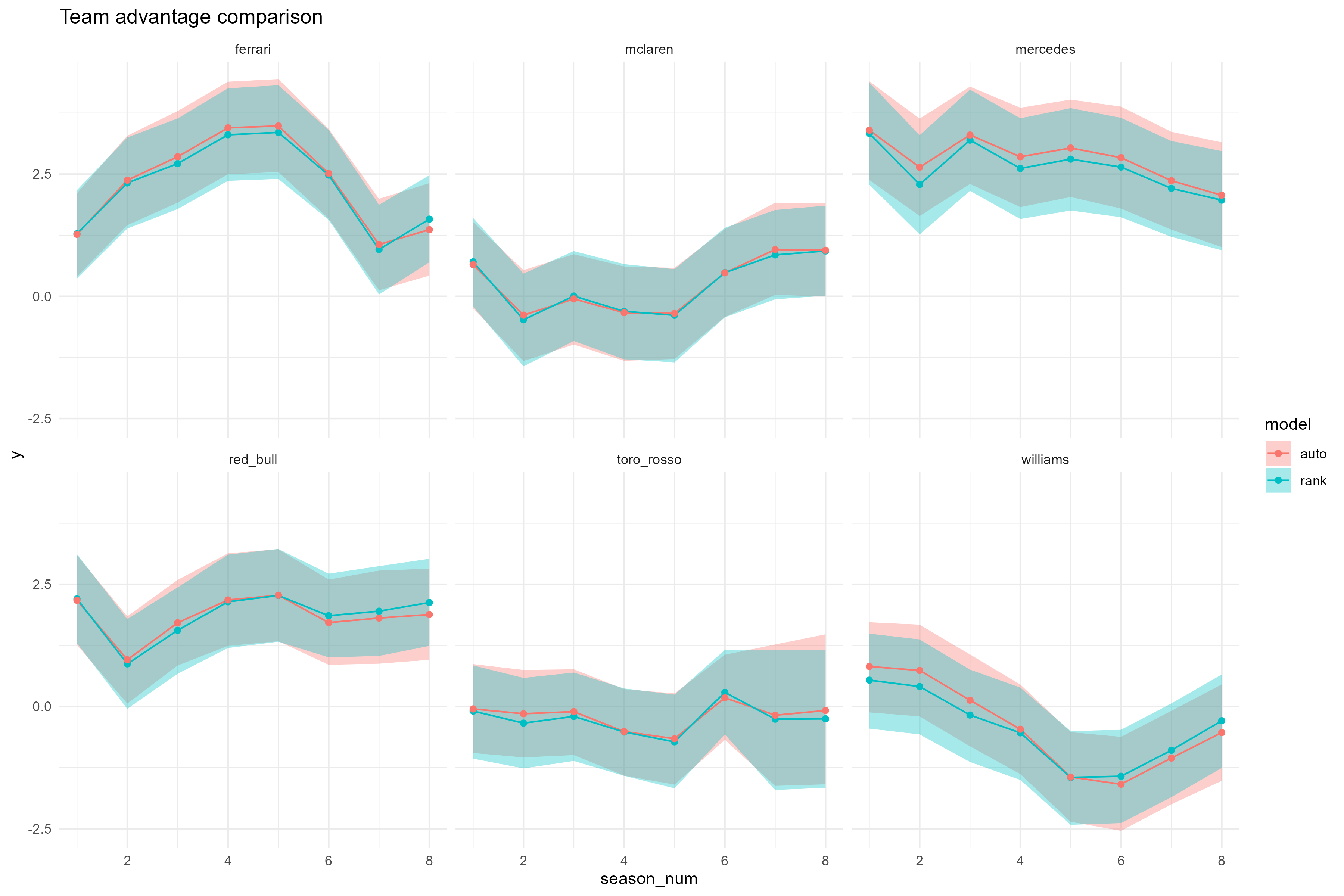

Supplement: Supplementary file 1 — Supplementary Material Details [file j_jqas-2022-0021_suppl_001.zip › model_comparison/img/ar_rank_team_comparison.png]

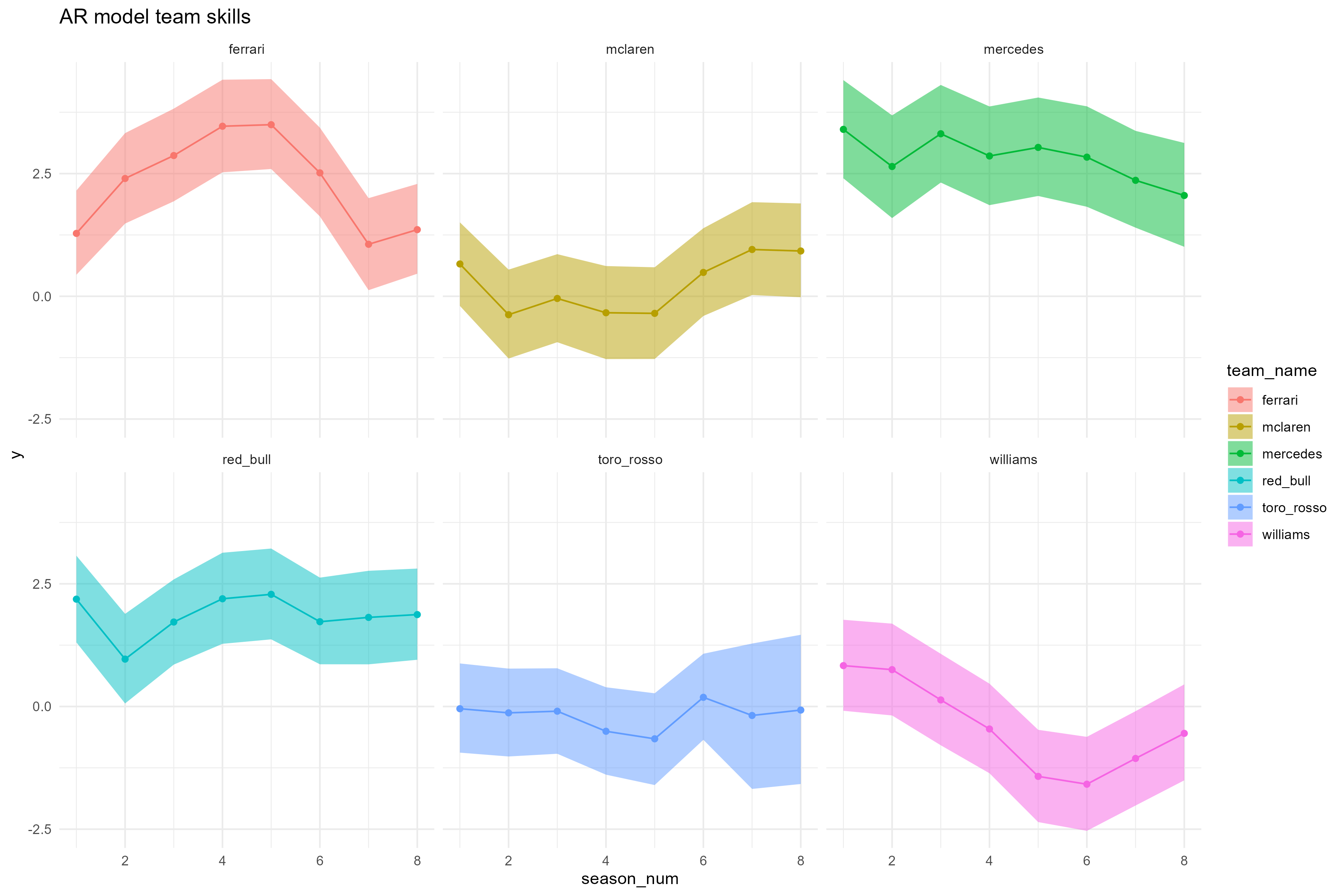

Supplement: Supplementary file 1 — Supplementary Material Details [file j_jqas-2022-0021_suppl_001.zip › model_comparison/img/ar_team.png]

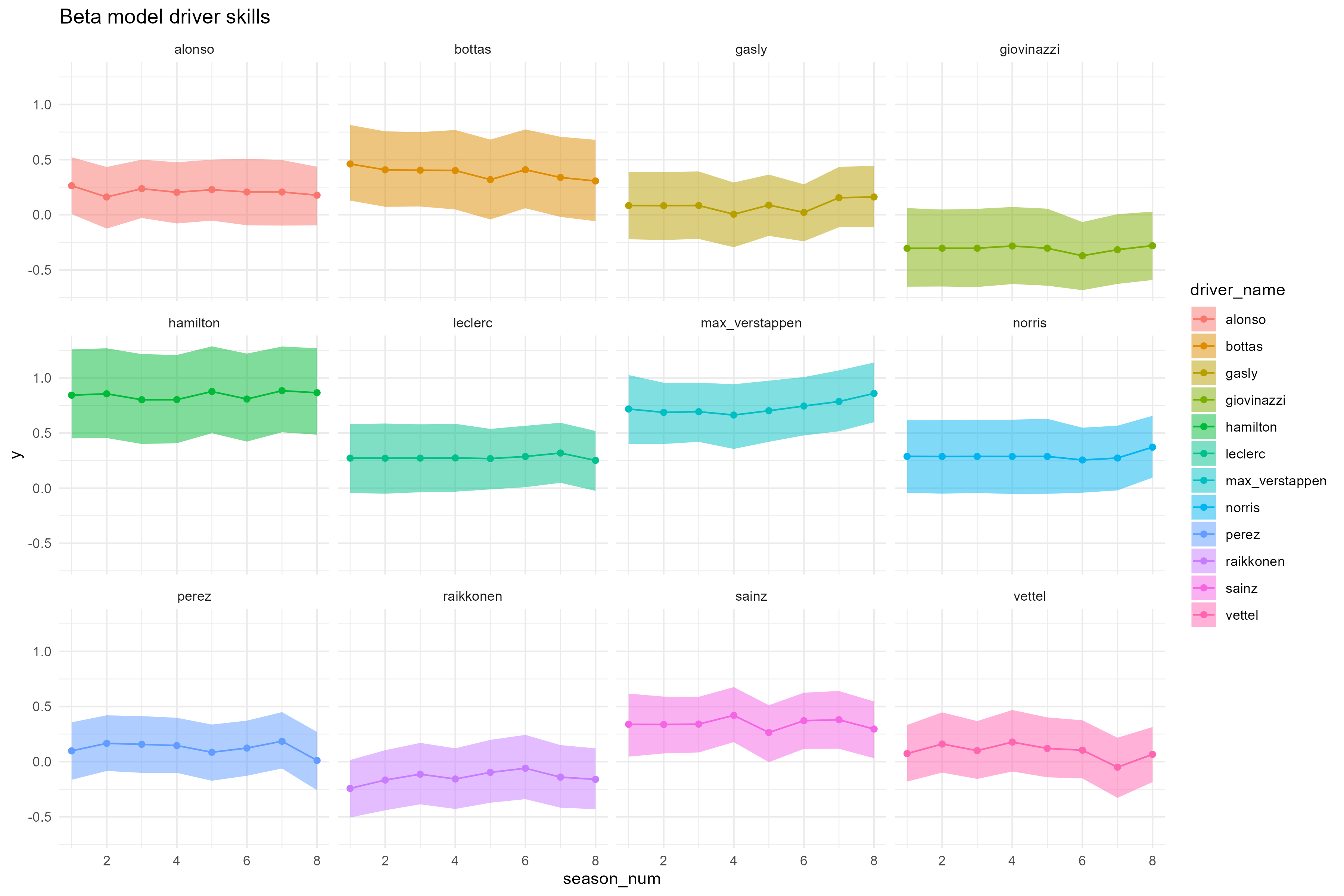

Supplement: Supplementary file 1 — Supplementary Material Details [file j_jqas-2022-0021_suppl_001.zip › model_comparison/img/beta_driver.png]

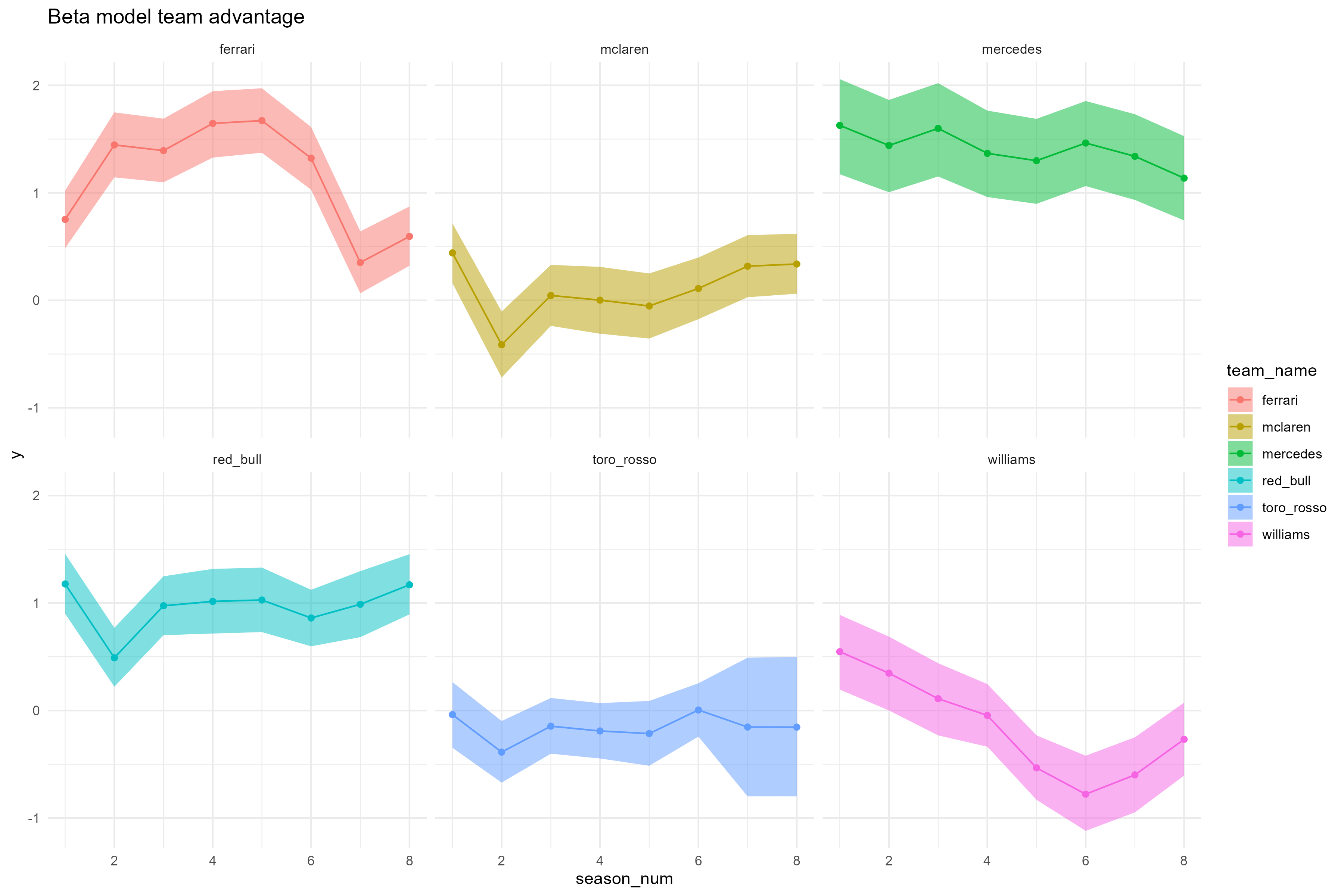

Supplement: Supplementary file 1 — Supplementary Material Details [file j_jqas-2022-0021_suppl_001.zip › model_comparison/img/beta_team.png]

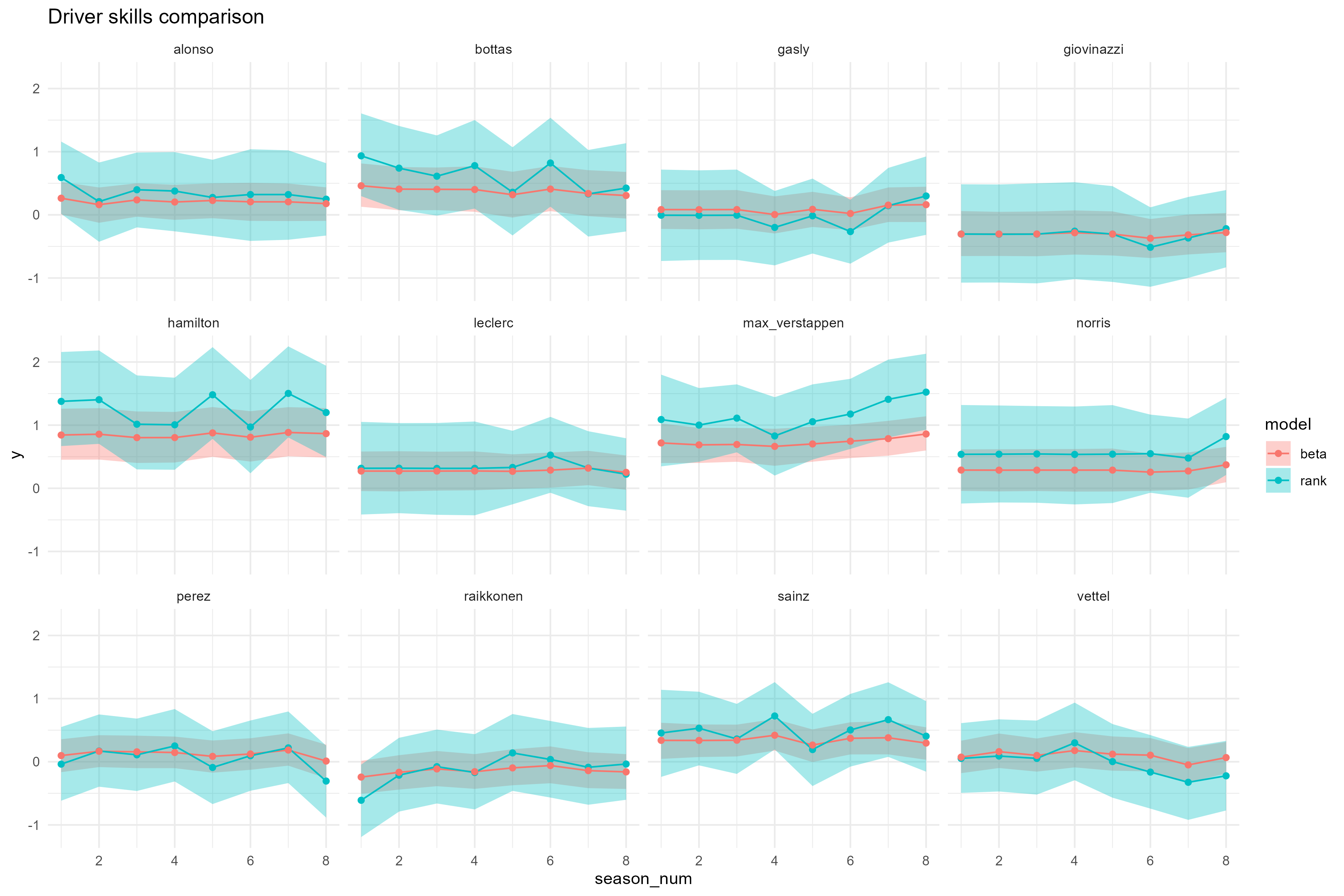

Supplement: Supplementary file 1 — Supplementary Material Details [file j_jqas-2022-0021_suppl_001.zip › model_comparison/img/driver_comparison.png]

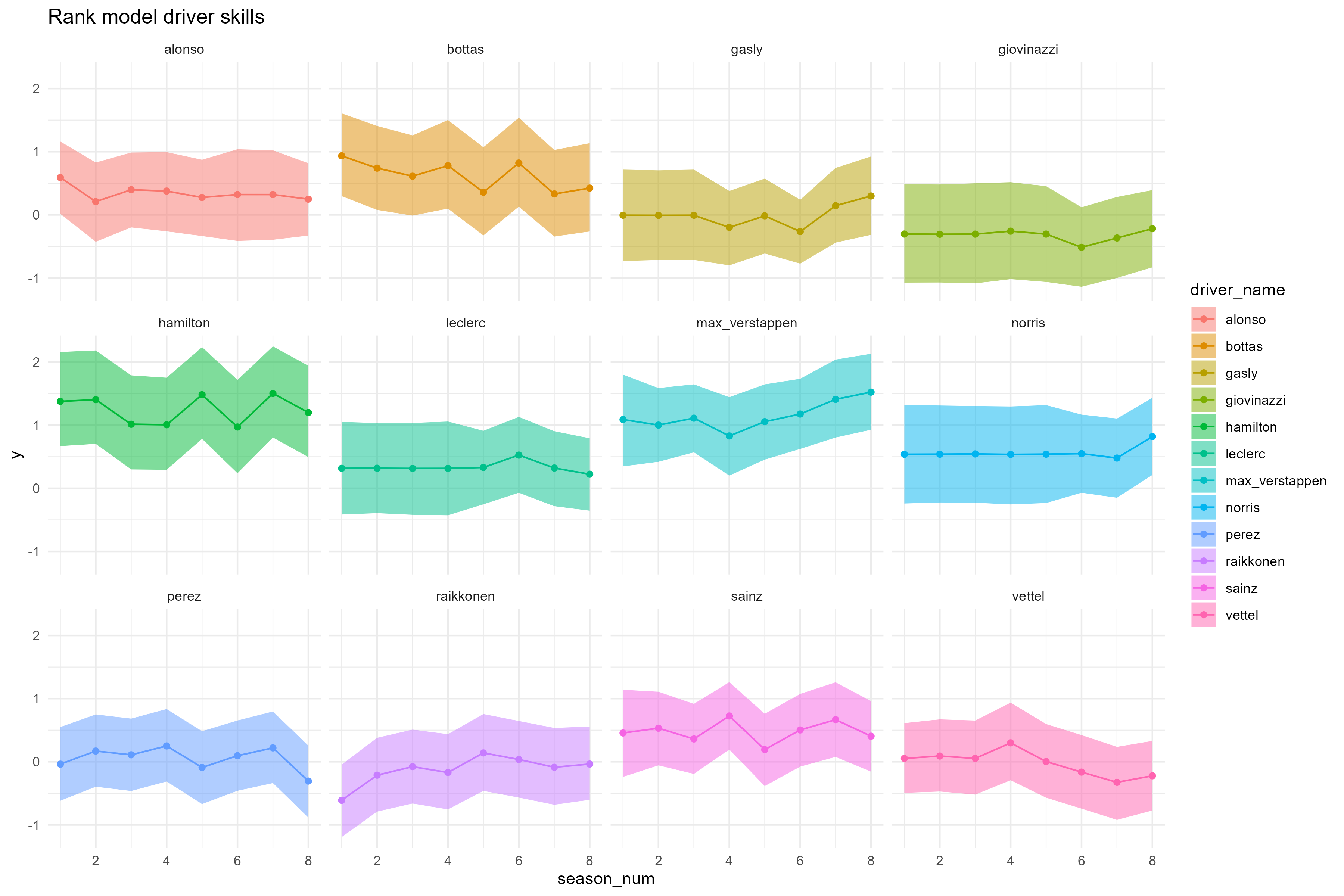

Supplement: Supplementary file 1 — Supplementary Material Details [file j_jqas-2022-0021_suppl_001.zip › model_comparison/img/rank_driver.png]

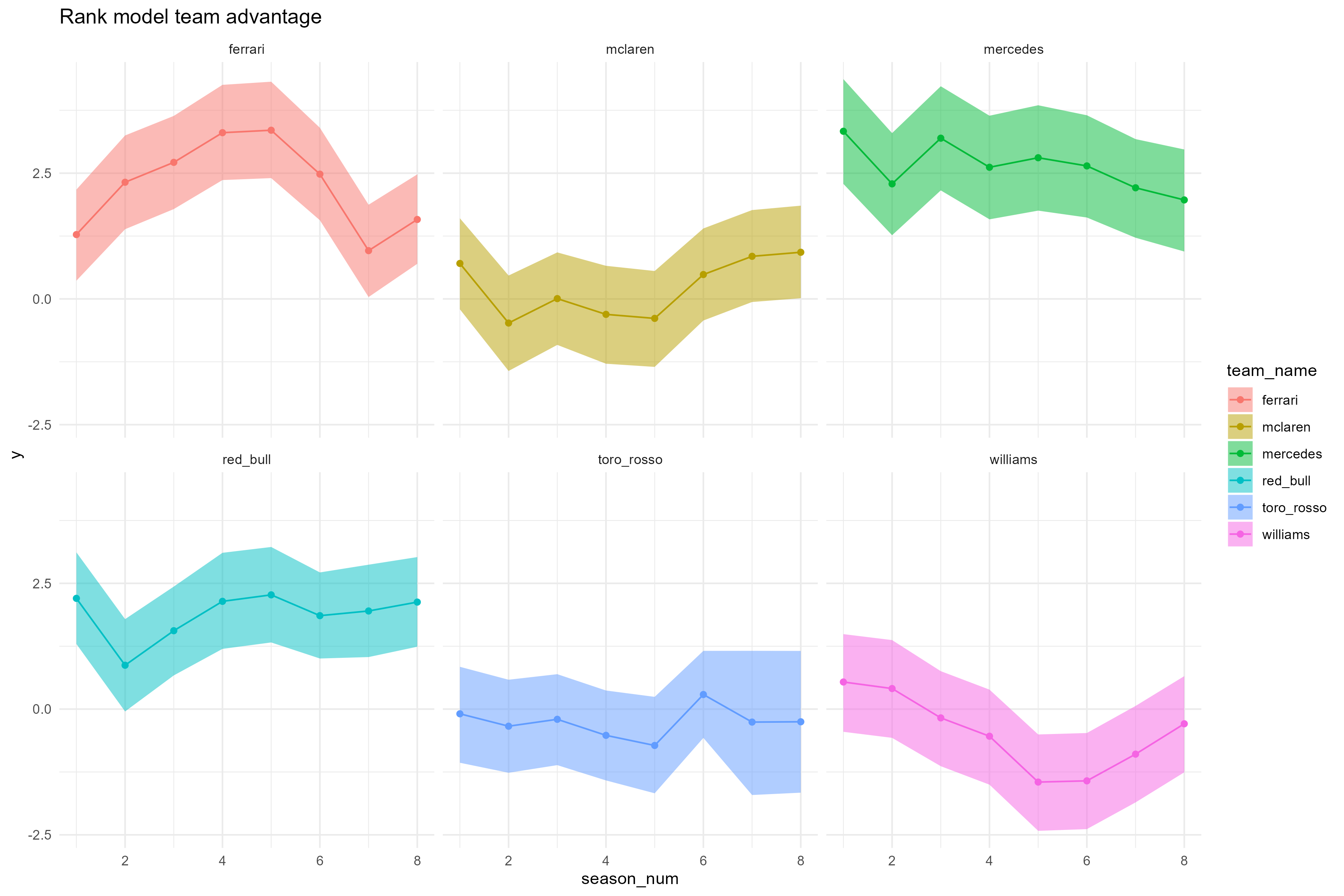

Supplement: Supplementary file 1 — Supplementary Material Details [file j_jqas-2022-0021_suppl_001.zip › model_comparison/img/rank_team.png]

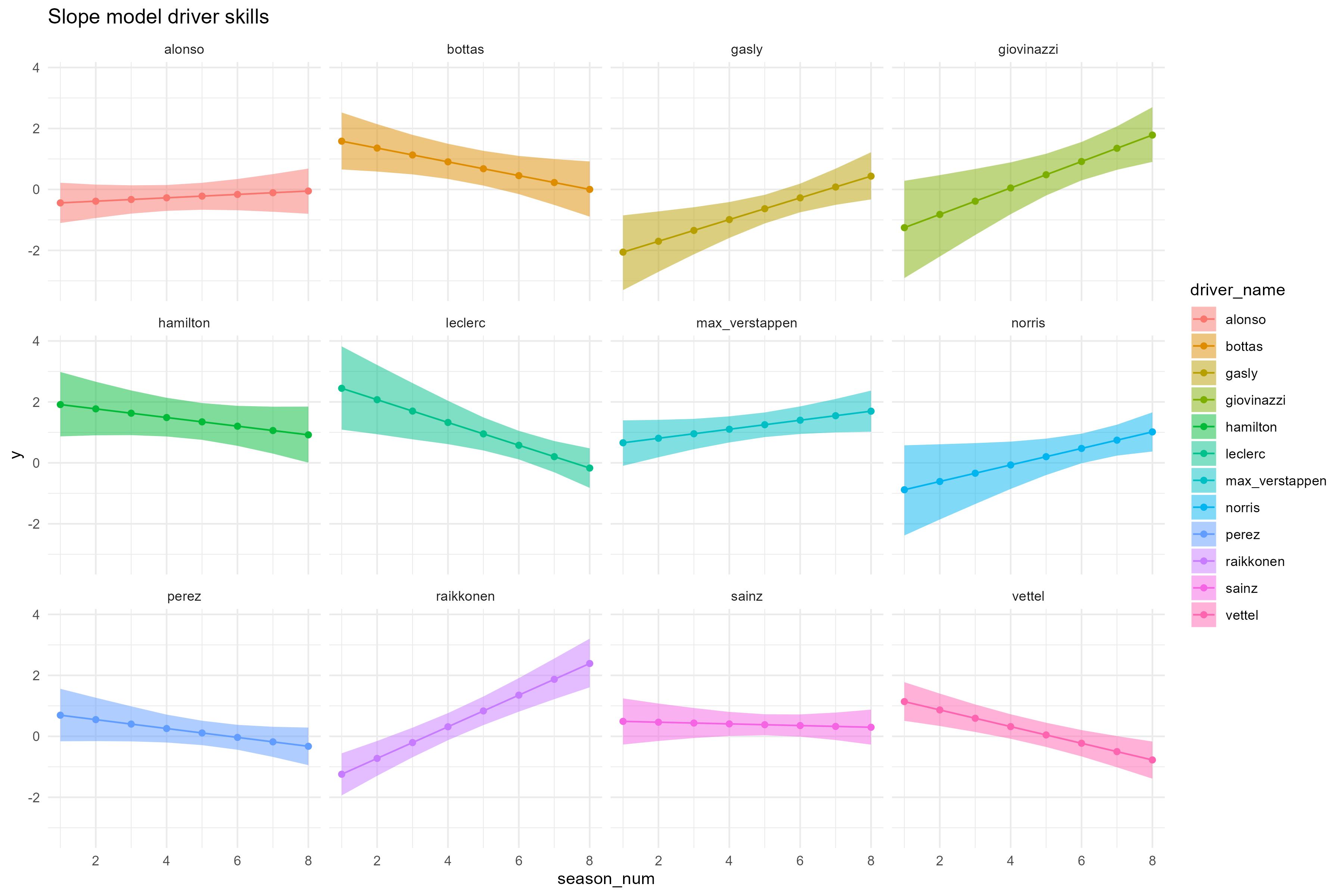

Supplement: Supplementary file 1 — Supplementary Material Details [file j_jqas-2022-0021_suppl_001.zip › model_comparison/img/slope_driver.png]

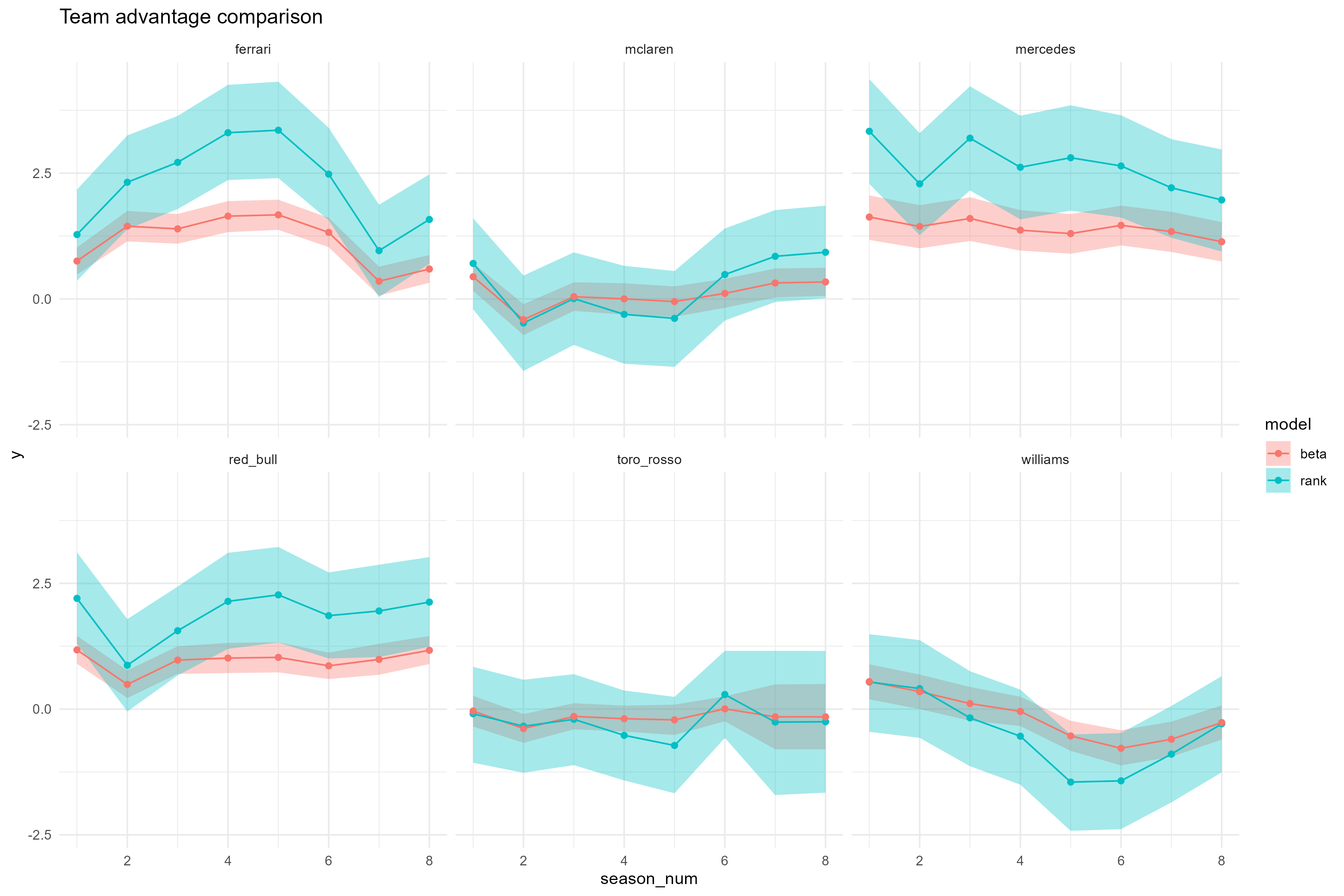

Supplement: Supplementary file 1 — Supplementary Material Details [file j_jqas-2022-0021_suppl_001.zip › model_comparison/img/team_comparison.png]

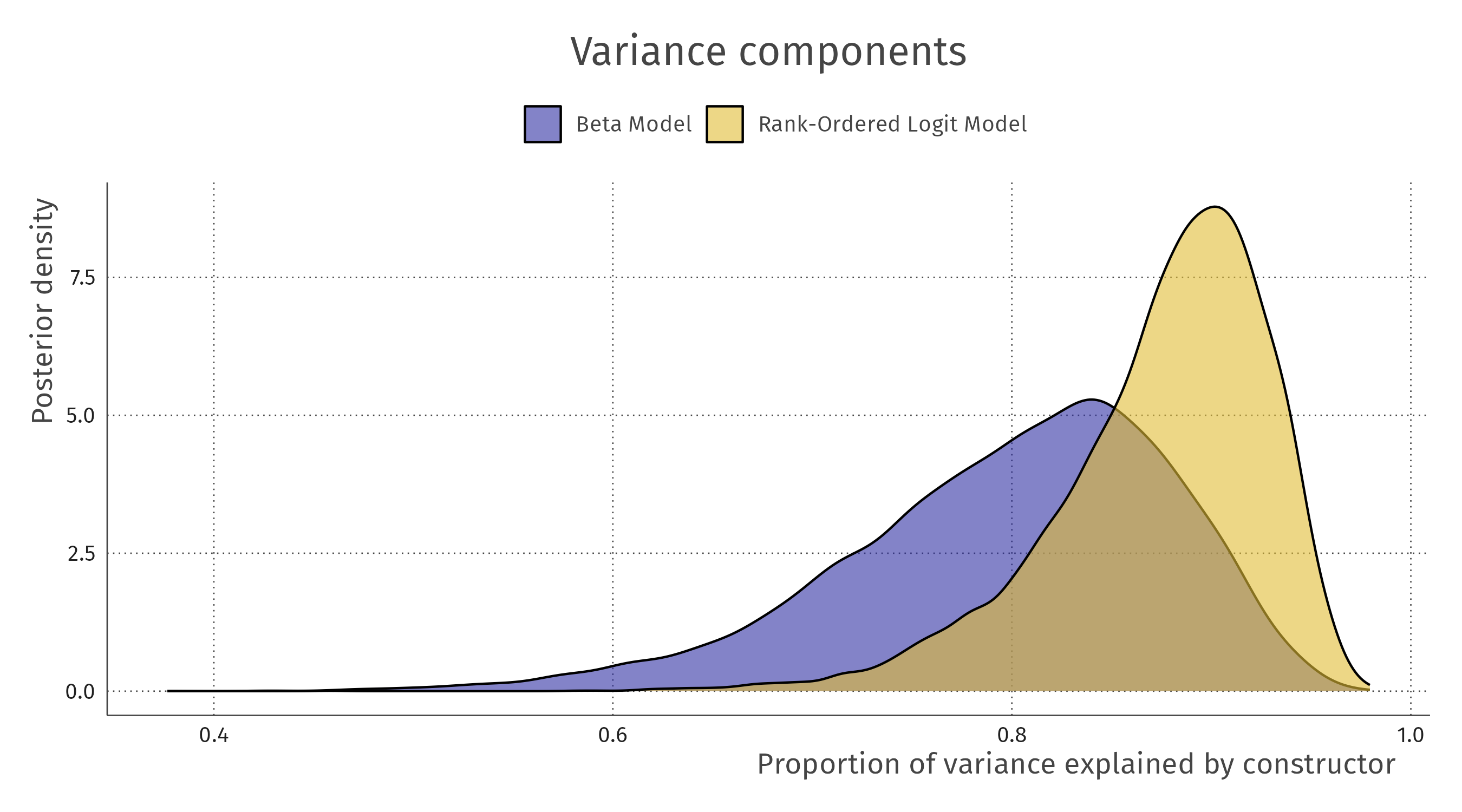

Supplement: Supplementary file 1 — Supplementary Material Details [file j_jqas-2022-0021_suppl_001.zip › model_comparison/img/variance.png]

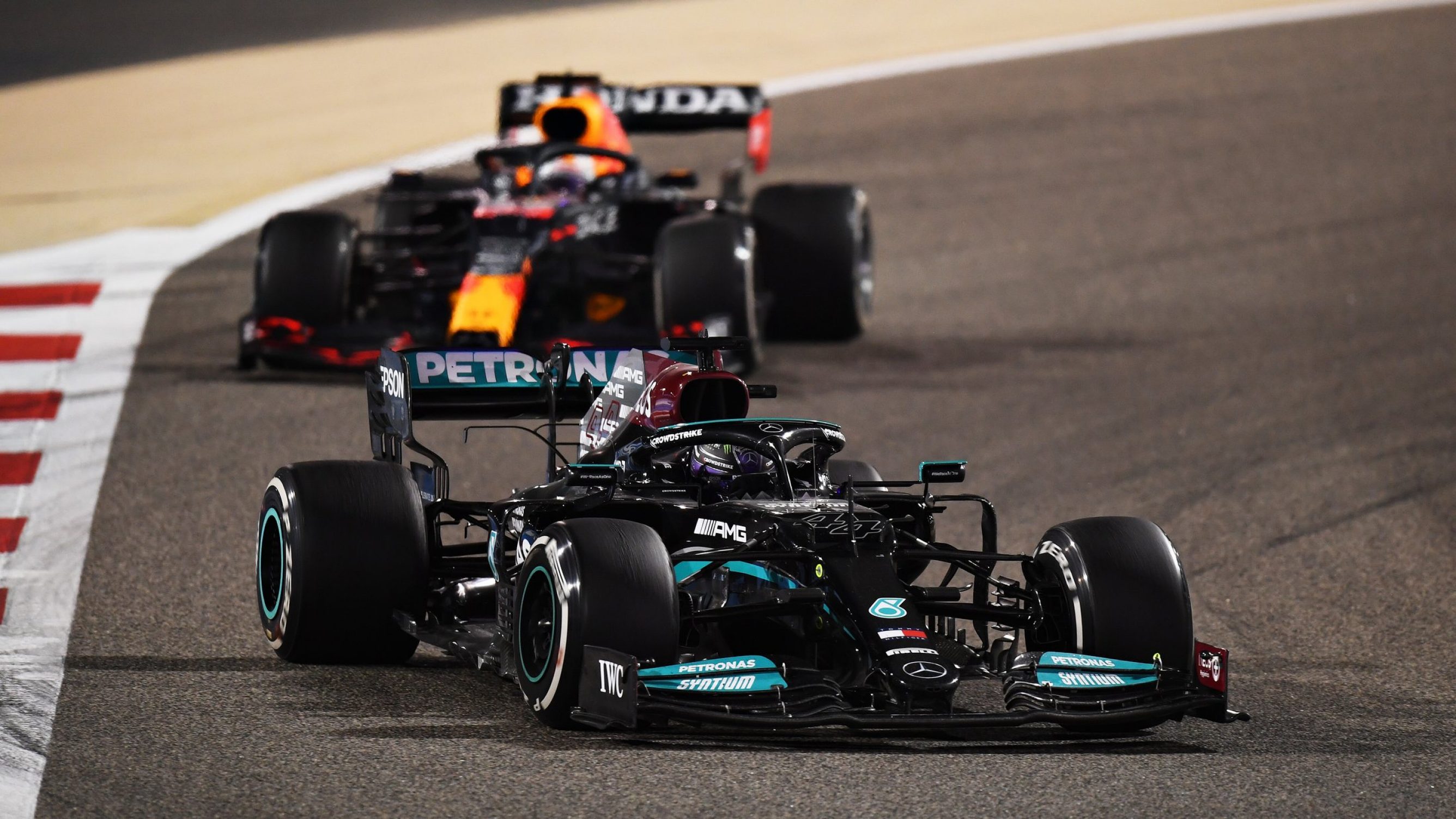

Supplement: Supplementary file 1 — Supplementary Material Details [file j_jqas-2022-0021_suppl_001.zip › pic/f1_battle.jpg]

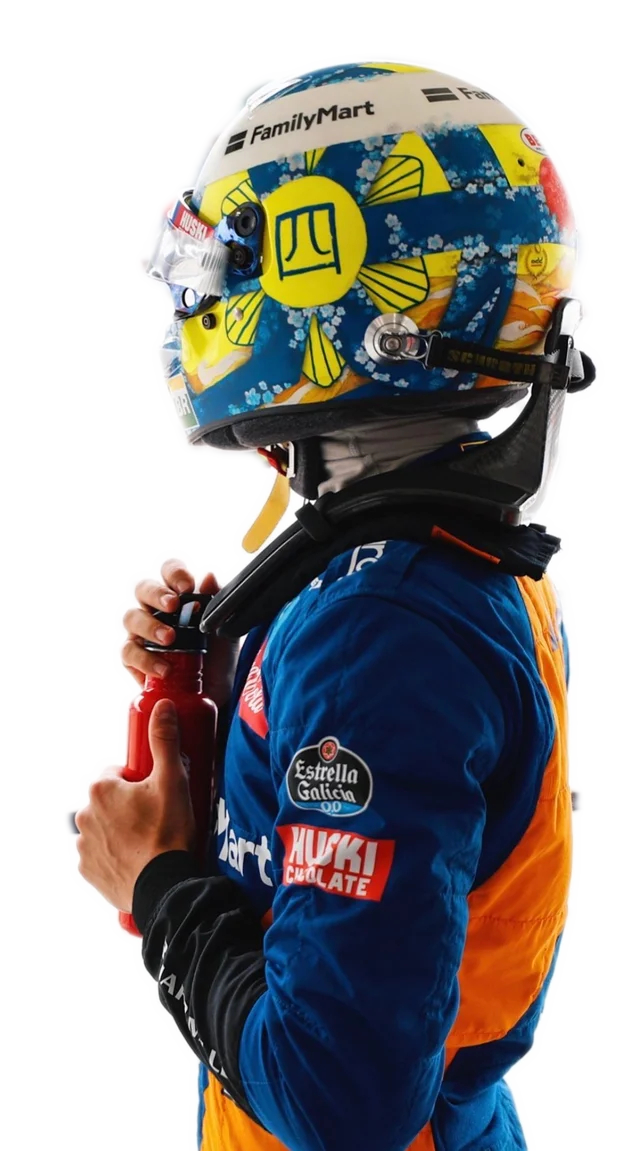

Supplement: Supplementary file 1 — Supplementary Material Details [file j_jqas-2022-0021_suppl_001.zip › pic/norris_cutout.png]

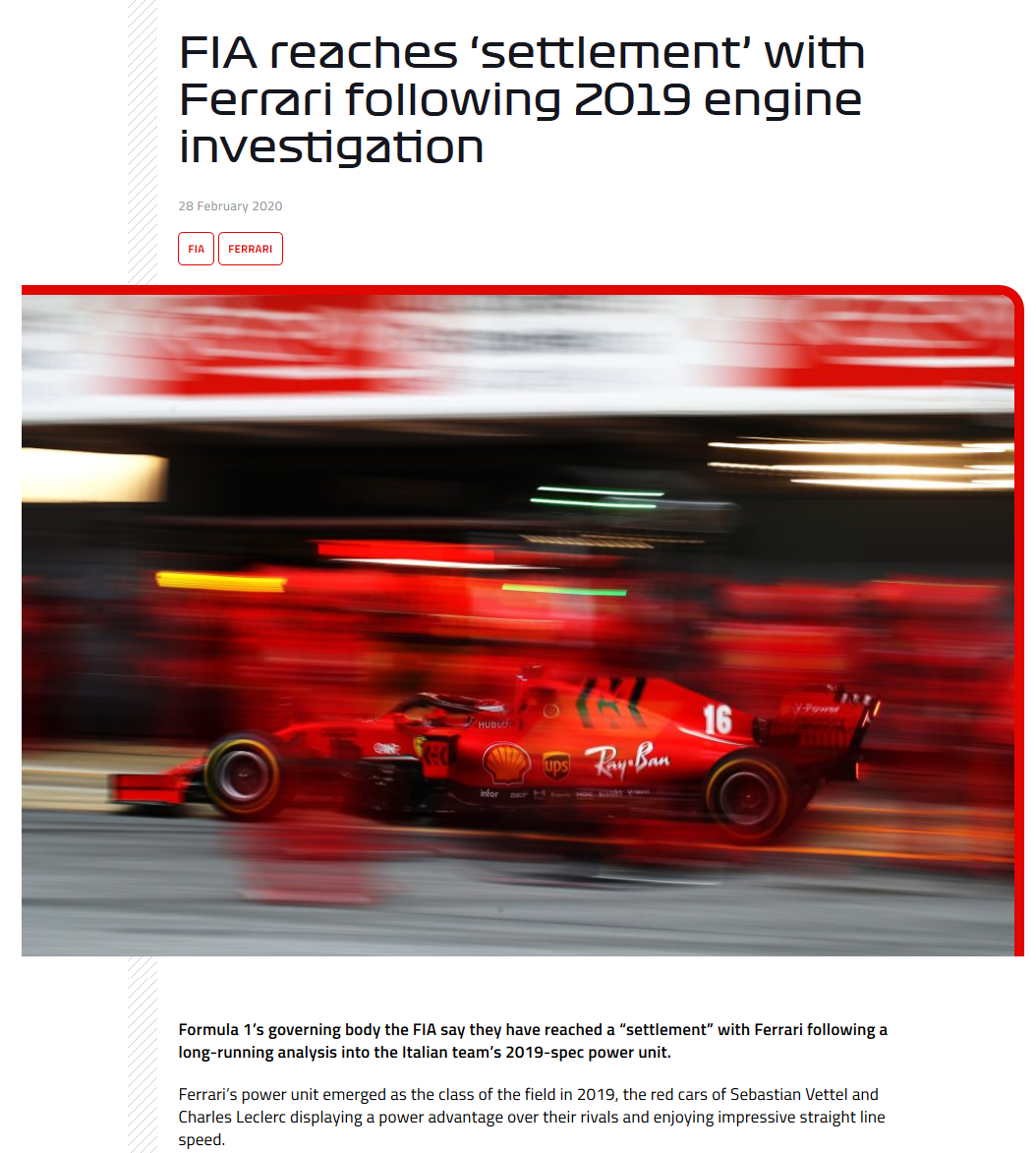

Supplement: Supplementary file 1 — Supplementary Material Details [file j_jqas-2022-0021_suppl_001.zip › pic/settlement.png]

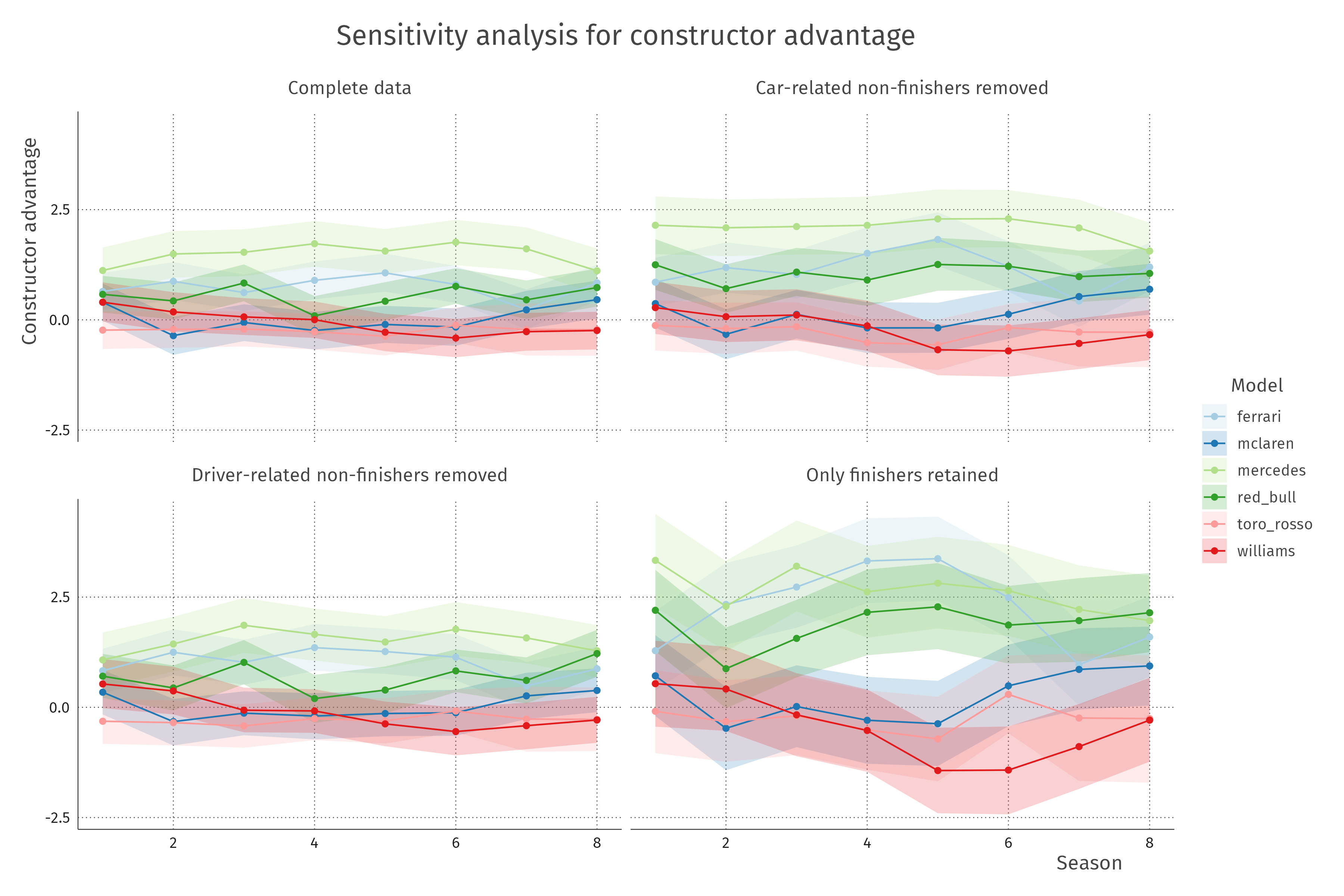

Supplement: Supplementary file 1 — Supplementary Material Details [file j_jqas-2022-0021_suppl_001.zip › sensitivity_analysis/img/constructor_advantage_comparison.png]

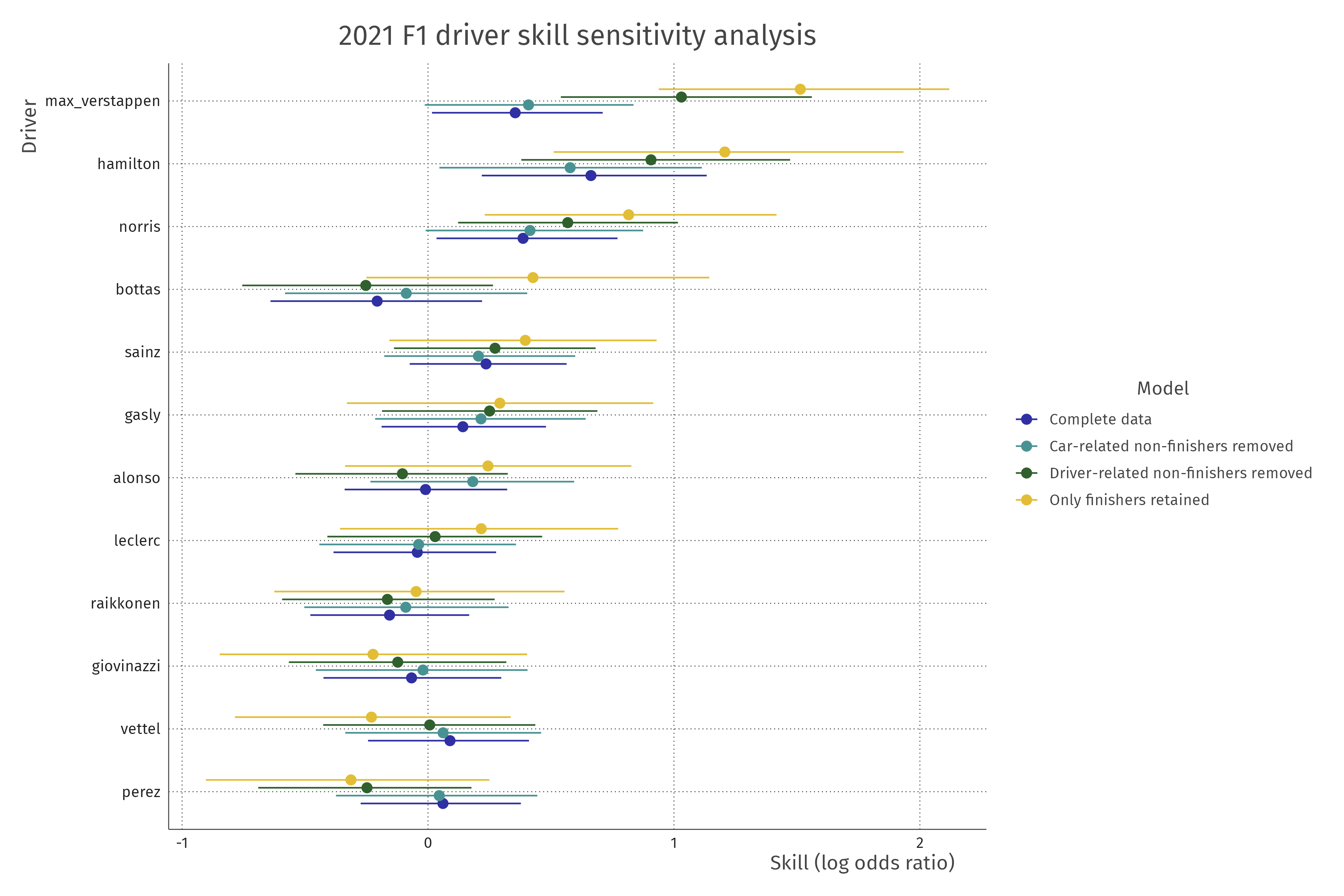

Supplement: Supplementary file 1 — Supplementary Material Details [file j_jqas-2022-0021_suppl_001.zip › sensitivity_analysis/img/driver_2021_comparison.png]

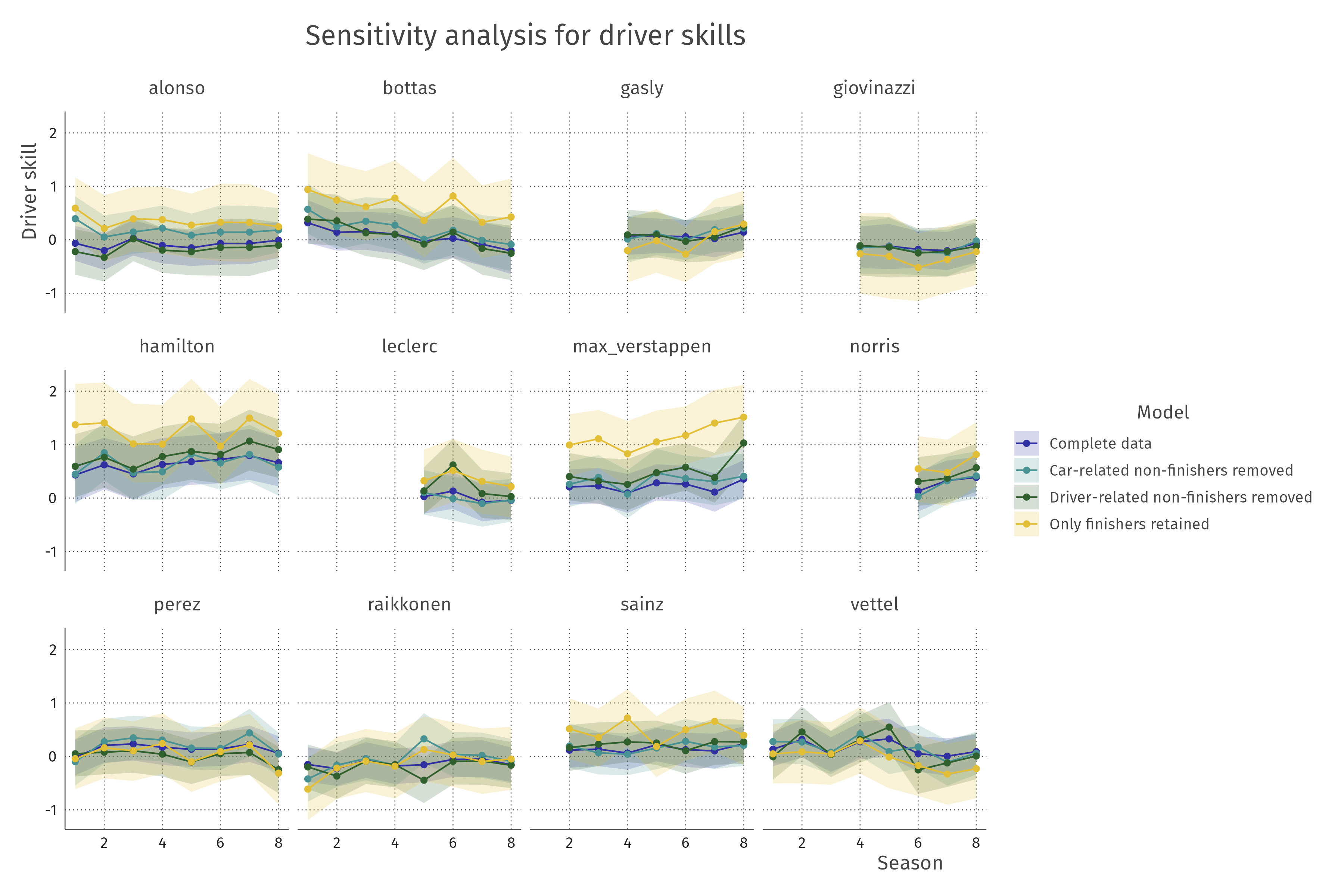

Supplement: Supplementary file 1 — Supplementary Material Details [file j_jqas-2022-0021_suppl_001.zip › sensitivity_analysis/img/driver_skills_comparison.png]
